# Explainable differential diagnosis with dual-inference large language models

**DOI:** 10.1038/s44401-025-00015-6

**Published:** 2025-04-24

**Authors:** Shuang Zhou, Mingquan Lin, Sirui Ding, Jiashuo Wang, Canyu Chen, Genevieve B. Melton, James Zou, Rui Zhang

**Affiliations:** 1 Division of Computational Health Sciences, Department of Surgery, University of Minnesota, Minneapolis, MN USA; 2 Bakar Computational Health Sciences Institute, University of California San Francisco, San Francisco, CA USA; 3 Department of Computer Science, University of Chicago, Chicago, IL USA; 4 Department of Computer Science, Illinois Institute of Technology, Chicago, IL USA; 5 Institute for Health Informatics and Division of Colon and Rectal Surgery, Department of Surgery, University of Minnesota, Minneapolis, MN USA; 6 Department of Biomedical Data Science, Stanford University, Stanford, CA USA

**Keywords:** Computational models, Translational research, Predictive medicine

## Abstract

Automatic differential diagnosis (DDx) involves identifying potential conditions that could explain a patient’s symptoms and its accurate interpretation is of substantial significance. While large language models (LLMs) have demonstrated remarkable diagnostic accuracy, their capability to generate high-quality DDx explanations remains underexplored, largely due to the absence of specialized evaluation datasets and the inherent challenges of complex reasoning in LLMs. Therefore, building a tailored dataset and developing novel methods to elicit LLMs for generating precise DDx explanations are worth exploring. We developed the first publicly available DDx dataset, comprising expert-derived explanations for 570 clinical notes, to evaluate DDx explanations. Meanwhile, we proposed a novel framework, Dual-Inf, that could effectively harness LLMs to generate high-quality DDx explanations. To the best of our knowledge, it is the first study to tailor LLMs for DDx explanation and comprehensively evaluate their explainability. Overall, our study bridges a critical gap in DDx explanation, enhancing clinical decision-making.

## Introduction

Differential diagnosis (DDx), a critical component of clinical care, involves generating a list of potential conditions that could explain a patient’s symptoms^1^. It facilitates comprehensive case evaluation, identifies critical but subtle conditions, guides diagnostic testing, and optimizes resource utilization. Additionally, DDx fosters patient involvement and trust through improved communication. While numerous automatic DDx systems^2,3^ have been developed to support decision-making, their black-box nature, particularly in deep learning models, often undermines trust^4^. To address this, providing interpretative insights alongside diagnostic predictions is essential^5^. Explainable DDx, which takes patient symptom descriptions as input, generates differential diagnoses, and offers accompanying explanations, is thus highly desirable in clinical practice.

In recent years, large language models (LLMs), such as ChatGPT, trained on extensive corpora, have exhibited remarkable capabilities in various clinical scenarios, including medical question answering (QA)^6–9^, clinical text summarization^10^, and disease diagnosis^11–16^. Motivated by these advancements, some studies have explored LLMs to improve diagnostic accuracy^17^. For instance, Daniel et al.^18^ fine-tuned PaLM 2 on medical data and developed an interactive interface to assist clinicians with DDx generation, while Savage et al.^19^ refined Chain-of-Thought (CoT) prompting^20^ to harness LLMs’ reasoning capabilities.

Despite these efforts, the potential of LLMs to generate reliable DDx explanations remains largely unexplored, leaving their role in supporting clinical decision-making uncertain. Two key challenges impede progress in this domain. First, the absence of DDx datasets annotated with diagnostic explanations limits model development and evaluation^21,22^. Second, numerous studies have highlighted LLMs’ inherent difficulties with complex reasoning tasks^23,24^, such as multi-step logical reasoning^25,26^ and clinical decision-making^27,28^. Thus, creating tailored datasets and developing novel methodologies to enable LLMs to synthesize high-quality DDx explanations are worthy of exploration.

In this study, we addressed these challenges by investigating prompting strategies for generating trustworthy DDx explanations. Our contributions are threefold. First, we curated a new dataset of 570 clinical notes across nine specialties, sourced from publicly available medical corpora and annotated by domain experts with differential diagnoses and explanations. To our knowledge, this is the first publicly available structured dataset with DDx explanation annotation^21,29^, which facilitates automated evaluation and holds substantial potential to advance the field. Second, we proposed Dual-Inf, a customized framework to optimize LLMs’ explanation generation capabilities. The core design lies in enabling LLMs to perform bidirectional inference (i.e., from symptoms to diagnoses and vice versa), leveraging backward verification to boost prediction correctness. Third, we comprehensively evaluated Dual-Inf for explainable DDx, including model explainability and error analysis. The results demonstrated that Dual-Inf achieved superior diagnostic performance while delivering reliable interpretations across various base LLMs (i.e., GPT-4, GPT-4o, Llama3-70B, and BioLlama3-70B). Overall, our findings highlight the effectiveness of Dual-Inf as a promising tool for improving clinical decision-making.

## Results

### Dataset

We developed Open-XDDx, a well-annotated dataset for explainable DDx, consisting of 570 clinical notes from publicly available medical exercises across nine specialties: cardiovascular, digestive, respiratory, endocrine, nervous, reproductive, circulatory, skin, and orthopedic diseases. Each note includes patient symptoms, differential diagnoses, and expert-derived explanations from the University of Minnesota (Supplementary Appendix 1). The dataset statistics are detailed in Table 1 and Table 2.

**Table 1:** The data characteristics of our annotated explainable DDx dataset Open-XDDx

Statistic	Value
Total number of notes	570
Mean note length (words)	113.6
Standard deviation of note length (words)	60.4
Mean number of diagnoses per note	4.6
Standard deviation of diagnoses per note	1.0
Mean number of explanations per patient	14.5
Standard deviation of explanations per patient	5.8
Mean number of explanations per diagnosis	3.1
Standard deviation of explanations per diagnosis	1.5

**Table 2:** Breakdown of the notes in the DDx dataset Open-XDDx across the nine clinical specialties

Clinical Specialty	Number of Notes (%)
Cardiovascular disease	26 (4.6%)
Digestive system disease	105 (18.4%)
Respiratory disease	58 (10.2%)
Endocrine disorder	43 (7.5%)
Nervous system disease	137 (24.0%)
Reproductive system disease	54 (9.5%)
Circulatory system disease	66 (11.6%)
Skin disease	30 (5.3%)
Orthopedic disease	51 (8.9%)

### Differential diagnosis performance

**Fig. 1:**
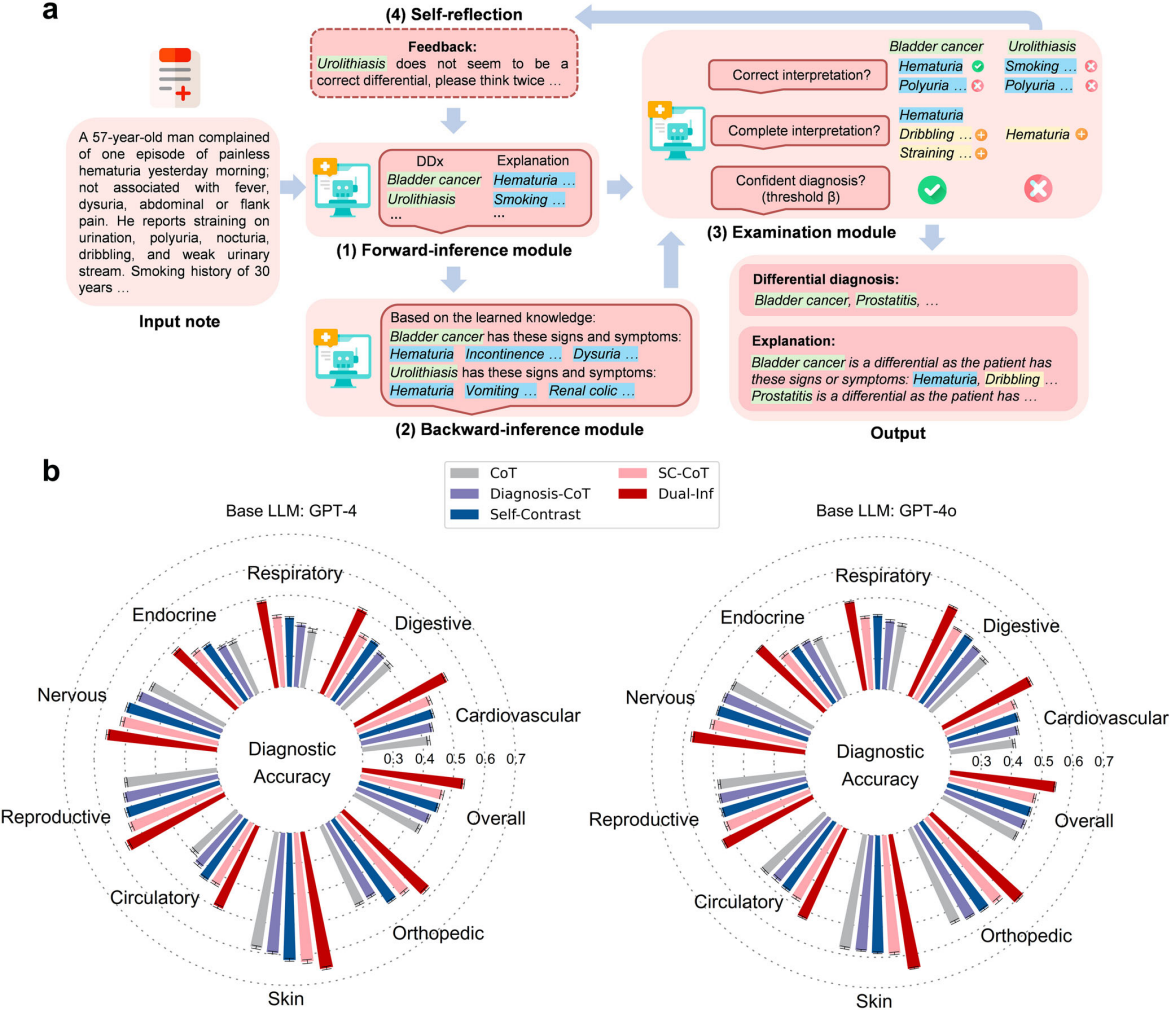
Overview of the proposed framework and differential diagnosis performance. **a** An overview of the Dual-Inference Large Language Model framework (Dual-Inf) for explainable DDx. Dual-Inf consists of four components: (1) a forward-inference module, which is an LLM to generate initial diagnoses from patient symptoms, (2) a backward-inference module, which is an LLM for conducting inverse inference via recalling all the representative symptoms associated with the initial diagnoses, i.e., from diagnoses to symptoms, (3) an examination module, which is another LLM to receive patients’ notes and the output from the two modules for prediction assessment (e.g., completeness examination) and decision making (e.g., filtering out low-confidence diagnoses), and (4) an iterative self-reflection mechanism, which iteratively takes the low-confidence diagnoses as feedback for the forward-inference module to “think twice”. **b** Differential diagnosis performance built on two base LLMs (GPT-4 and GPT-4o) over nine specialties. The results are averaged over five runs. Standard deviations are also shown.

We evaluated differential diagnosis accuracy (Eq. 1) by comparing model predictions to ground-truth diagnoses with prompts (Supplementary Appendix 3). The results with GPT-4 and GPT-4o are depicted in Fig. 1(b), and the results with Llama3-70B and BioLlama3-70B are presented in Supplementary Appendix 4. It showed that Dual-Inf consistently outperformed baselines across nine specialties. Specifically, when built on GPT-4, the overall performance of SC-CoT significantly exceeded CoT (difference of 0.032, 95% CI 0.021–0.043, *p* = 0.001) and Diagnosis-CoT (difference of 0.019, 95% CI 0.001–0.028, *p* = 0.004). Dual-Inf further surpassed SC-CoT (0.533 vs. 0.472, difference of 0.061, 95% CI 0.055–0.062, *p* < 0.001). Concretely, the performance improvement of Dual-Inf over SC-CoT exceeded 16% on cardiovascular and digestive diseases. Similarly, using GPT-4o, Dual-Inf achieved over 0.55 accuracy on nervous, skin, and orthopedic diseases, exceeding the baselines by over 9%. With Llama3-70B and BioLlama3-70B, Dual-Inf outperformed SC-CoT by over 10% in cardiovascular, digestive, and respiratory diseases. The overall performance improvement of Dual-Inf over SC-CoT across the three base LLMs (difference of 0.059, 0.048, and 0.049) was statistically significant (*p* < 0.001).

### Interpretation performance

**Fig. 2:**
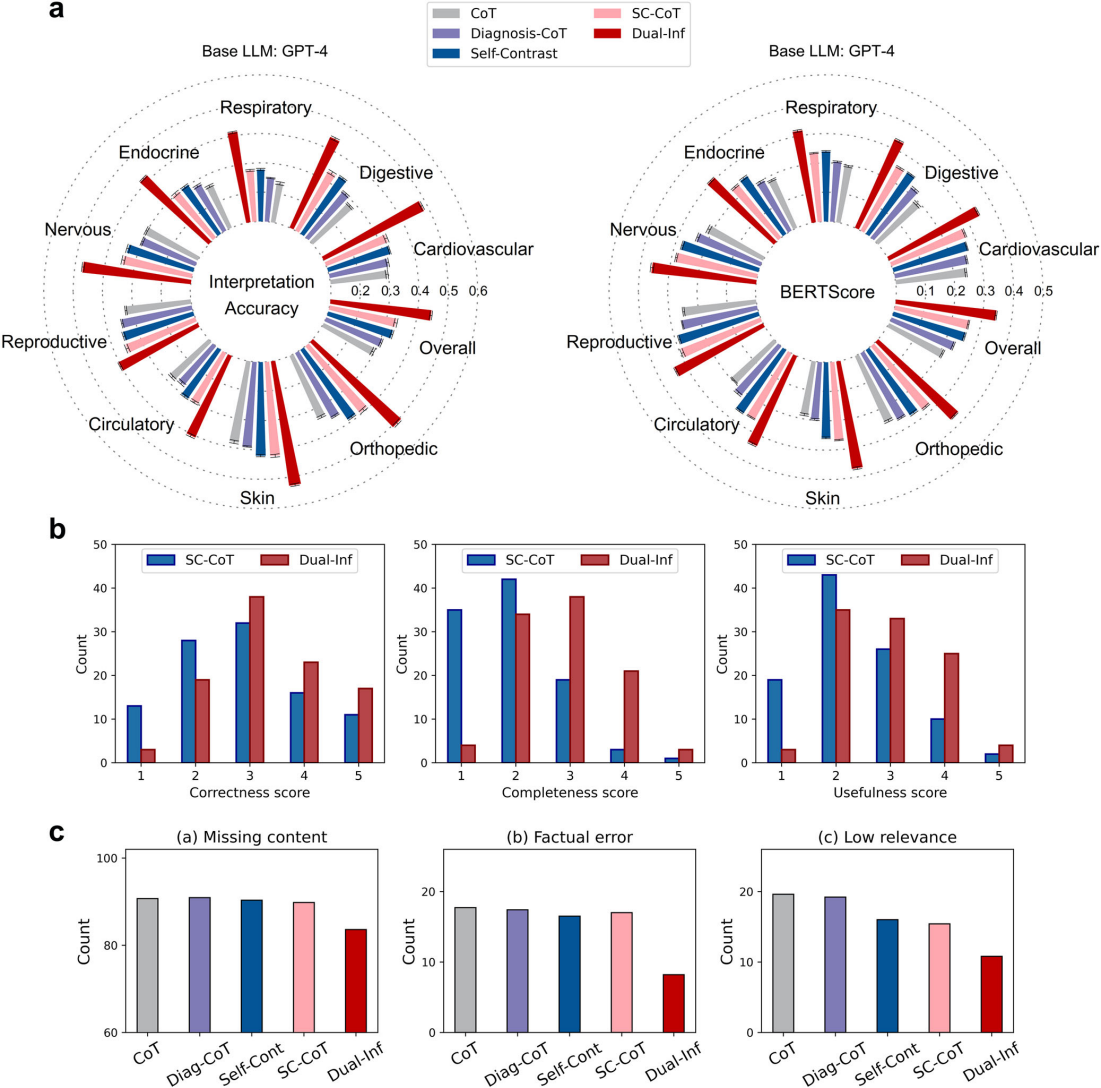
Interpretation performance and error analysis. **a** Interpretation performance w.r.t interpretation accuracy (see Eq. 2) and BERTScore across nine clinical specialties. We implemented the methods with GPT-4. The results were averaged over five runs. Standard deviations were also shown. **b** Human evaluation results on interpretation. It assessed three aspects: correctness, completeness, and usefulness, with scores ranging from 1 to 5. **c** Error type analysis on interpretation. We manually examined 100 cases and recorded the count of the error type. Diag-CoT denotes Diagnosis-CoT, and Self-Cont means Self-Contrast. The results were averaged over five runs. The methods are implemented with GPT-4.

Model explainability was examined through automatic and human assessments. For automatic evaluation, GPT-4o was employed to measure the consistency between ground-truth and predicted interpretations, utilizing prompts detailed in Supplementary Appendix 3. We tested four base LLMs (GPT-4, GPT-4o, Llama3-70B, and BioLlama3-70B). Partial results on GPT-4 are shown in Fig. 2(a), with additional results in Supplementary Appendix 5. In Fig. 2(a), the interpretation accuracy (Eq. 2) of Diagnosis-CoT and SC-CoT was 0.305 and 0.334, surpassing CoT by 0.011 (95% CI 0.004–0.019, *p* = 0.012) and 0.04 (95% CI 0.038–0.043, *p* < 0.001), respectively. Dual-Inf achieved even higher accuracy at 0.446, with a 0.112 improvement over SC-CoT (95% CI 0.105–0.118, *p* < 0.001). Concretely, the performance improvement of Dual-inf over the baselines surpassed 26% in cardiovascular and respiratory diseases. For BERTScore, SentenceBert, and METEOR, Dual-Inf outperformed SC-CoT with comparisons of 0.345 vs. 0.258 (difference of 0.087, 95% CI 0.083–0.090, *p* < 0.001), 0.427 vs. 0.356 (difference of 0.071, 95% CI 0.067–0.076) and 0.333 vs. 0.251 (difference of 0.082, 95% CI 0.076–0.088). When taking GPT-4o as the base LLM, the interpretation accuracy of Dual-Inf reached 0.488, outperforming CoT and SC-CoT, which scored 0.366 and 0.408, respectively. On the other metrics, Dual-Inf consistently surpassed SC-CoT, with differences of 0.083, 0.064, and 0.08. Similarly, with Llama3-70B and BioLlama3-70B, Dual-Inf exceeded SC-CoT by over 17% across all metrics. In detail, the performance improvement on digestive, respiratory, and endocrine diseases exceeded 25% over the baselines w.r.t interpretation accuracy (Supplementary Appendix 5).

The interpretations were also manually examined by clinicians on three qualitative metrics: Correctness, Completeness, and Usefulness (Supplementary Appendix 2). Figure 2b presents the results for 100 randomly selected notes, using GPT-4 as the base LLM. We observed that the Correctness score of Dual-Inf predominantly ranged from 3 to 4, whereas SC-CoT scores mainly fell between 2 and 3. In terms of Completeness score, Dual-Inf achieved 38 scores of 3 and 21 scores of 4, compared to SC-CoT’s 19 and 3, respectively. Regarding the Usefulness score, Dual-Inf had 33 scores of 3 and 25 scores of 4, while SC-CoT had 26 and 10, respectively.

### Case study

**Fig. 3:**
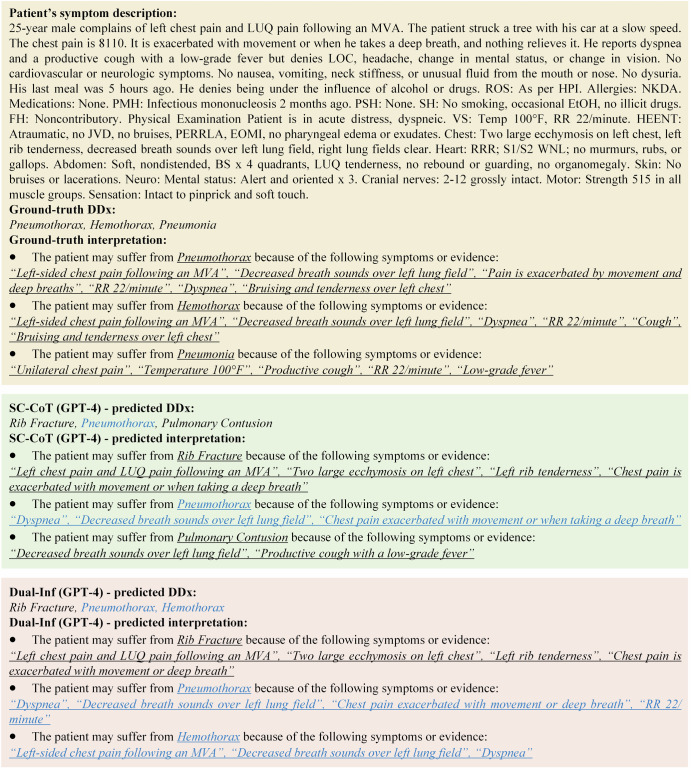
Case study of SC-CoT and Dual-Inf. The methods are implemented by taking GPT-4 as the base LLM. Correct predictions are highlighted in blue.

We further provided case studies to demonstrate the superior explainability of Dual-Inf over the baselines. The example in Fig. 3 showcased that SC-CoT only provided three correct explanations for a differential, i.e., *Pneumothorax*, while Dual-Inf generated more accurate explanations. Besides, Dual-Inf had one more correct differential, i.e., *Hemothorax*, with three correct explanations than the baselines. See more examples and detailed illustrations in Supplementary Appendices 7 and 8.

### Error analysis on explanation

We analyzed error types in generated explanations by comparing Dual-Inf with the baselines on 100 randomly selected samples with incorrect outputs. Errors were categorized as missing content (missing at least two pieces of evidence), factual errors (medically incorrect), or low relevance (evidence not highly pertinent) based on prior studies^30,31^. Using GPT-4 as the base LLM (Fig. 2(c)), SC-CoT had 89 cases of missing content versus 76 for Dual-Inf (difference 13.4, 95% CI 11.5–15.2). For factual errors, the baselines achieved similar performance, and the count number comparison between Dual-Inf and SC-CoT was 17 vs. 8.2 (difference 8.8, 95% CI 7.8–9.8). As for low-relevance, Self-Contrast and SC-CoT had fewer errors than the CoT and Diagnosis-CoT, while the comparison between SC-CoT and Dual-Inf was 15.4 vs. 10.8 (difference 4.6, 95% CI 3.9–5.3). All differences were statistically significant (*p* < 0.001). We further presented the count of errors in each clinical specialty in Supplementary Appendix 6. The results demonstrated that the errors fell into all the specialties, while the nervous and digestive diseases specialty had more errors.

### Ablation study

We evaluated the contribution of each component in Dual-Inf through four variants: (1) forward-inference only (FI), (2) FI with excluded backward-inference (FI-EM), (3) FI-EM without self-reflection (FI-EM*), and (4) Dual-Inf without self-reflection (Dual-Inf*). Specifically, we adopted automatic metrics for the evaluation. The results in Supplementary Appendix 12 confirmed that Dual-Inf achieved superior diagnostic accuracy and explainability, highlighting the necessity of all components.

## Discussion

**Fig. 4:**
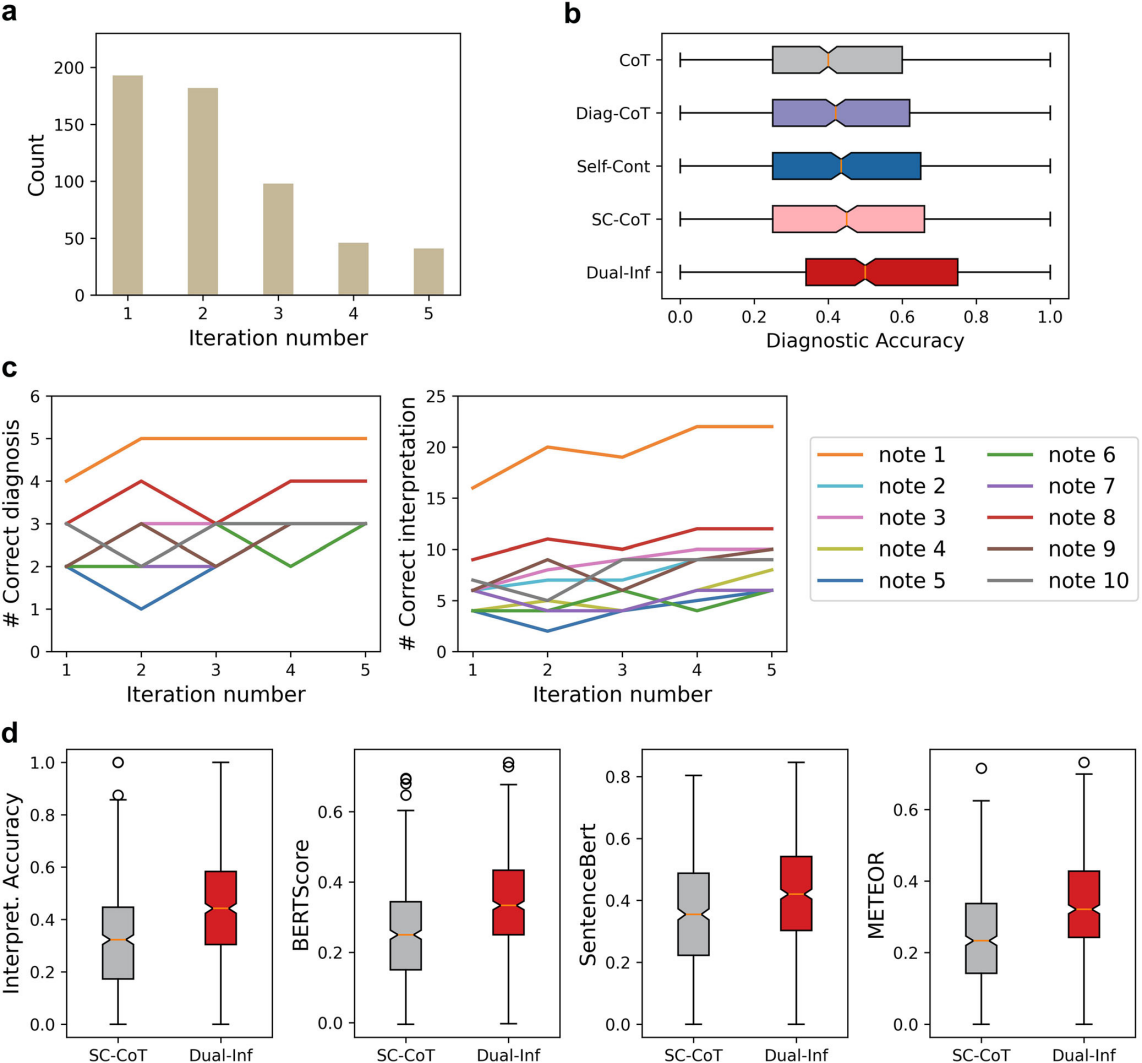
In-depth analysis of Dual-Inf. **a** Data statistics of the iteration number for each note in Dual-Inf. **b** Distribution visualization of diagnostic accuracy on each note. Diag-CoT denotes Diagnosis-CoT, and Self-Cont means Self-Contrast. **c** Performance change on Dual-Inf w.r.t diagnosis and explanation after each iteration. We randomly selected ten notes with five iterations. In this figure, the base LLM of the methods is GPT-4. **d** Distribution visualization of interpretation performance on each note. SC-CoT and Dual-Inf were implemented with GPT-4. The circular points shown as outliers mean that some scores are deviated from the vast majority.

Our study demonstrated that Dual-Inf significantly enhanced diagnostic accuracy by filtering low-confidence diagnoses through quality assessment. Specifically, the examination module consolidated outputs from other components to verify correctness, while the self-reflection mechanism enabled the forward-inference module to refine predictions iteratively. To evaluate iterative reflection, we tracked the iteration count for each note in Dual-Inf (Fig. 4a), revealing that most predictions were iteratively revised. For randomly selected ten notes with five iterations, the number of correct diagnoses improved progressively (Fig. 4c), confirming the effectiveness of the iterative reflection mechanism. Besides, we observed that most of the notes’ prediction correctness was boosted or remained stable in the fourth or fifth iteration, demonstrating the necessity of setting the maximum iteration λ to a relatively large value (e.g., 5). Additionally, the distribution of diagnostic accuracy across cases, visualized in Fig. 4b, showed that the median and upper quartile for Dual-Inf (0.495 and 0.746) outperformed SC-CoT (0.434 and 0.652) and Diagnosis-CoT (0.421 and 0.628), with statistically significant improvements (*p* < 0.001). These findings highlight the efficacy of Dual-Inf in enhancing diagnostic accuracy.

Second, Dual-Inf produced superior DDx explanations. Manual evaluation of 100 cases (Fig. 2b) showed higher scores across all metrics, attributed to bidirectional inferences and iterative prediction refinement. Iterative reflection effectiveness was confirmed through ten notes with five iterations (Fig. 4c) and a case study of intermediate predictions (Supplementary Appendix 7), both demonstrating improved explanations over iterations. Distribution analysis (Fig. 4d) revealed higher median and quartile scores (e.g., BERTScore, METEOR) for Dual-Inf compared to SC-CoT, confirming its ability to generate better explanations across most cases. Notably, although the note snippets were publicly available, the ground-truth of DDx and the corresponding explanations were manually generated by our domain experts. Therefore, the LLMs have not been exposed to the ground-truth, and the evaluation on our dataset is trustworthy.

Third, this study demonstrated that leveraging multiple LLMs mitigates explanation errors in DDx. As shown in Fig. 2c, Self-Contrast and SC-CoT reduced low-relevance errors compared to CoT and Diagnosis-CoT, highlighting the benefit of integrating multiple LLM interpretations to address hallucinations. Dual-Inf further minimized errors across all the types, attributed to its dual-inference scheme: the forward-inference module generated diagnoses, the backward-inference module recalled medical knowledge, and the examination module refined predictions. The self-reflection mechanism further improved explanation quality and reduced hallucinations through iterative refinement. Additionally, the higher error rates observed in the nervous and digestive disease specialties were attributed to their larger sample sizes in the dataset. However, normalizing error counts by sample size revealed comparable error rates across the specialties.

One limitation of this study is that our dataset, encompassing nine clinical specialties, does not fully capture the breadth of real-world scenarios. Besides, the dataset lacks annotations on the priority of each diagnosis within the DDx, as ranking the likelihood of possible diseases presents significant challenges. Furthermore, the backward-inference module’s reliance on internal medical knowledge to generate reference signs and symptoms makes it vulnerable to severe hallucinations or erroneous knowledge, which could impact performance. This issue can be mitigated by implementing Dual-Inf with advanced LLMs^32^.

In summary, this study established a manually annotated dataset for explainable DDx and designed a tailored framework that effectively harnessed LLMs to generate high-quality explanations. The findings revealed that existing prompting methods exhibited suboptimal performance in generating DDx and explanations, limiting their practical utility in clinical scenarios. Our experiments verified the effectiveness of Dual-Inf for providing accurate DDx, delivering comprehensive explanations, and reducing prediction errors. Furthermore, the released dataset with ground-truth DDx and explanations could facilitate the research field. Future work could expand the dataset to a broader range of clinical specialties or integrate domain knowledge from external databases for superior performance.

## Methods

### Data acquisition and processing

The data source is publicly available medical exercises collected from medical books^33,34^ and MedQA USMLE dataset^35^. There are two key criteria for selecting the clinical notes: (1) the notes must originate from disease diagnosis exercises; (2) they must pertain to one of the nine clinical specialties. We transformed the exercises into free text by preserving the symptom descriptions and removing the multiple-choice options, where applicable. The texts were further preprocessed, including (1) removing duplicate notes, (2) unifying all characters into UTF-8 encoding and removing illegal UTF-8 strings, (3) correcting or removing special characters, and (4) filtering out notes with fewer than 130 characters. Lastly, we collected 570 clinical notes, among which 10 notes were used for prompt development, and 560 notes were preserved for evaluation. The full dataset can be found in Supplementary Appendix 13.

### Data annotation

The raw data generally did not have the annotation of DDx, explanation, and clinical specialty. To build a well-annotated dataset, we employed three clinical physicians to curate the dataset manually. Two independent physicians annotated each exercise. When disagreement existed in the annotation, a third physician examined the case and made the final annotation. We checked the inter-annotator agreement (IAA) on DDx, interpretation, and specialty (Supplementary Appendix 1). Additionally, our dataset is well-structured in a standardized format, facilitating automated evaluation.

### Model development

How to effectively elicit LLMs’ capability for accurate DDx explanation is challenging. Inspired by the fact that humans usually conduct backward reasoning to validate the correctness of answers when solving reasoning problems^36^, we proposed performing backward verification (i.e., from diagnosis to symptoms) to examine the predicted diagnoses and elicit correct answers via self-reflection. Accordingly, we developed a customized framework called Dual-Inference Large Language Model (Dual-Inf), shown in Fig. 1a. Specifically, Dual-Inf consisted of four components: (1) a forward-inference module, which was an LLM for initial diagnoses, i.e., from patients’ symptoms to diagnoses, (2) a backward-inference module, which was an LLM for inverse inference via recalling all the representative symptoms of the initial diagnoses, i.e., from diagnoses to symptoms, (3) an examination module, which was another LLM that received patients’ notes and the output from the two modules for prediction assessment and decision making, and (4) an iterative self-reflection mechanism, which iteratively took low-confidence diagnoses as feedback for the forward-inference module to “think twice”.

The pipeline was as follows. First, the forward-inference module analyzed clinical notes to infer initial diagnoses and provide interpretations. Next, the backward-inference module received the initial diagnoses as input and recalled the representative symptoms that the diagnoses generally present, including medical examination and laboratory test results. Given that the recalled symptoms were derived from the LLM’s internal knowledge, they are generally reliable in advanced LLMs^30,32^ and could serve as a reference for measuring the correctness of the predicted explanations. Afterward, the examination module verified and refined the above results. Specifically, it (*i*) checked the forward-inference module’s explanations against the recalled knowledge and discarded erroneous ones, (*ii*) supplemented the interpretations by integrating patient notes with recalled knowledge, (*iii*) decided whether to accept or filter predictions based on their quality. The underlying idea was that a diagnosis supported by fewer interpretations was deemed less trustworthy. To assess diagnostic confidence, a threshold *β* was applied: diagnoses with fewer than *β* supporting interpretations were flagged as low-confidence. Later, the self-reflection mechanism took the low-confidence diagnoses as feedback to prompt the forward-inference module to “think twice.” This iterative process continued up to a maximum limit λ, balancing accuracy with efficiency. Upon reaching this limit, the framework outputted the final results. The prompts for the three modules are detailed in Supplementary Appendix 9. Importantly, the prompts for the forward-inference module were carefully designed to ensure objectivity toward feedback from the examination module, reducing the risk of false negatives undermining correct predictions.

### Implementation details

We adopted four baselines: (1) CoT^20^, a popular prompting method; (2) Diagnosis-CoT^19^, a customized prompting method for disease diagnosis; (3) Self-Contrast^37^, an advanced method with multiple prompts and a re-examination mechanism to enhance reasoning; (4) Self-consistency CoT (SC-CoT)^38^, which assembled multiple reasoning paths to enhance performance. We followed the original papers in the implementation. Specifically, SC-CoT generated five reasoning paths for each note and then selected the most consistent diagnoses and interpretations. The prompts of baselines were shown in Supplementary Appendix 10. As for Dual-Inf, we incorporated CoT into the three LLM-based modules. The maximum iteration number *λ* was assigned to 5, considering the trade-off between effectiveness and efficiency; the threshold *β* was set to 3. We further analyzed the impact of the hyper-parameter *β* on the performance and presented the results in Supplementary Appendix 11. For a fair comparison, all the methods were implemented with the same base LLM, including GPT-4, GPT-4o, Llama3-70B (https://huggingface.co/meta-llama/Meta-Llama-3-70B), and BioLlama3-70B (https://huggingface.co/aaditya/Llama3-OpenBioLLM-70B). For the former two LLMs, we used the API from the OpenAI company (https://platform.openai.com/docs/models), which were “gpt-4-turbo-preview” and “gpt-4o”; for the latter two, we downloaded the models from Huggingface for inference. The temperature parameter was set as 0.1.

### Performance evaluation

We conducted automatic evaluation by comparing the ground-truths with the predicted ones. Following related papers^27^, we used accuracy as the primary metric for assessing diagnostic performance, i.e.,

1 $${\rm{Diagnostic\; Accuracy}}=\frac{{\rm{Cumulative\; number\; of\; correct\; diagnoses}}}{{\rm{Total\; number\; of\; diagnoses}}}$$

For interpretation performance, we employed metrics designed to assess the semantic alignment between the reference text and the predicted text, rather than relying solely on string matching. The metrics, including accuracy, BERTScore^39^, SentenceBert^40^, and METEOR^41^, have been widely used in related tasks^42,43^. Concretely, interpretation accuracy was computed as:

2 $${\rm{Interpretation\; Accuracy}}=\frac{{\rm{Cumulative\; number\; of\; correct\; interpretations}}}{{\rm{Total\; number\; of\; interpretations}}}$$

BERTScore^39^ employs the BERT model^44^ to determine the semantic similarity between reference and generated text, offering a context-aware evaluation of model performance. SentenceBert^40^ measures sentence similarity using a BERT model that generates dense vector representations, facilitating efficient and accurate semantic comparisons. METEOR^41^ assesses the harmonic mean of unigram precision and recall, utilizing stemmed forms and synonym equivalence. The details of automatic and human evaluation are shown in Supplementary Appendix 2.

## Supplementary information

Supplementary Appendix 13 - Full dataset

Supplementary Appendix_R1

## Data Availability

Data is provided in the supplementary information files.

## Code availability

The code used for this study is available at https://github.com/betterzhou/Dual-Inf.
