# Supplementary material for: Explainable differential diagnosis with dual-inference large language models
Source: Npj Health Syst. 2025 Apr 24;2:12. doi: 10.1038/s44401-025-00015-6 (PMC12021655; doi:10.1038/s44401-025-00015-6)
Supplement: Supplementary file 2 — Supplementary Appendix_R1 [file 44401_2025_15_MOESM2_ESM.pdf]

Supplementary Appendix 1

Annotation Guideline

This annotation guideline is for the Explainable Differential Diagnosis (DDx) task.

Background

Differential diagnosis (DDx) is a list of possible conditions that could cause a patient's symptoms. In practice, merely providing diagnosis predictions to clinicians is still not enough, as the accuracy of the predictions cannot be guaranteed, making them hard to trust. It is crucial to further explain the reasons behind the diagnoses to enhance their trustworthiness. Thereby, explainable DDx is desired in clinical scenarios. This guideline describes the specific types of information that should be annotated for explainable DDx.

Definition

Disease

A disease is a particular abnormal condition that adversely affects the structure or function of all or part of an organism and is not immediately due to any external injury. Diseases are often known to be medical conditions that are associated with specific signs and symptoms. In many cases, terms such as disease, disorder, morbidity, sickness, and illness are used interchangeably.

Differential Diagnosis

A differential diagnosis (DDx) is a systematic method used in medicine to generate a comprehensive list of potential conditions that could explain a patient's symptoms and signs, aiding in accurate diagnosis and treatment planning.

Sign and Symptom

A medical sign is an objective observable indication of a disease, injury, or medical condition that may be detected during a physical examination. These signs may be visible, such as a rash or bruise, or otherwise detectable such as by using a stethoscope or taking blood pressure. A symptom is something out of the ordinary that is experienced by an individual such as feeling feverish, a headache, or other pains in the body.

Medical Test

A medical test is a medical procedure performed to detect, diagnose, or monitor diseases, disease processes, susceptibility, or to determine a course of treatment. Medical tests such as physical and visual exams, diagnostic imaging, genetic testing, chemical and cellular analysis, relating to clinical chemistry and molecular diagnostics, are typically performed in a medical setting.

DDx Interpretation

DDx interpretation denotes the corresponding reasons or explanations that supported the potential diagnosis. The reasons for the diagnosis are based on the patient's signs, symptoms, and medical test results in the patient's clinical note.

Clinical Specialty

A clinical specialty is a branch of medical practice that is focused on a defined group of patients, diseases, skills, or philosophy. Here, we introduce 9 clinical specialties, including cardiovascular disease, digestive system disease, respiratory disease, endocrine disorder, nervous system disease, reproductive system disease, circulatory system disease, skin disease, and orthopedic disease. Please note that we only list some representative diseases to introduce the definition of the clinical specialties.

1) Cardiovascular disease
   Cardiovascular disease (CVD) is a cluster of diseases involving the heart or heart-related blood vessels. It is worth noting that in our paper, cardiovascular diseases are separated from circulatory system diseases and designated as a distinct department. This is because cardiovascular diseases form a relatively cohesive group on their own, and they pose significant health risks to humans, with a generally higher level of concern and awareness among people. We present some examples of CVDs as follows: coronary artery diseases (e.g., angina, heart attack), heart failure, hypertensive heart disease, rheumatic heart disease, cardiomyopathy, arrhythmia, congenital heart disease, valvular heart disease, and carditis.

2) Digestive system disease
   Digestive system disease is any disease involving the organs of the digestive system, which includes the gastrointestinal tract and accessory organs. Digestive system diseases constitute a class of diseases that include: esophageal diseases (e.g., gastroesophageal reflux disease, Barretts esophagus), stomach diseases (e.g., gastritis, peptic ulcers), intestinal diseases (e.g., Crohns disease, ulcerative colitis, irritable bowel syndrome), liver diseases (e.g., hepatitis, cirrhosis), pancreatic diseases (e.g., pancreatitis, pancreatic cancer), gallbladder diseases (e.g., cholecystitis, gallstones), and other disorders affecting the function of these organs.

3) Respiratory disease
   Respiratory disease is any disease involving the organs and tissues of the respiratory system, which includes the airways, lungs, and respiratory muscles. Respiratory diseases constitute a class of diseases that include: obstructive lung diseases (e.g., chronic obstructive pulmonary disease (COPD), asthma), restrictive lung diseases (e.g., pulmonary fibrosis, sarcoidosis), respiratory infections (e.g., pneumonia, tuberculosis), lung cancer, pleural diseases (e.g., pleurisy, pleural effusion), sleep-related breathing disorders (e.g., sleep apnea), pulmonary vascular diseases (e.g., pulmonary hypertension, pulmonary embolism), and conditions affecting the respiratory muscles (e.g., myasthenia gravis, muscular dystrophy).

4) Endocrine disorder

Endocrine disorder is any disease involving the endocrine glands and the hormones they produce. Endocrine disorders constitute a class of diseases that include: thyroid diseases (e.g., hypothyroidism, hyperthyroidism, thyroid cancer), adrenal gland diseases (e.g., Addisons disease, Cushings syndrome), pituitary gland diseases (e.g., pituitary adenomas, hypopituitarism), pancreatic endocrine diseases (e.g., diabetes mellitus, insulinoma), parathyroid diseases (e.g., hyperparathyroidism, hypoparathyroidism), gonadal disorders (e.g., hypogonadism), and other conditions affecting the hormonal balance and function of these glands.

5) Nervous system disease
Nervous system disease is any disease involving the central or peripheral nervous systems. Nervous system diseases constitute a class of diseases that include: neurodegenerative diseases (e.g., Alzheimers disease, Parkinsons disease), demyelinating disea ses (e.g., multiple sclerosis), cerebrovascular diseases (e.g., stroke), peripheral neuropathies (e.g., diabetic neuropathy, Guillain-Barré syndrome), seizure disorders (e.g., epilepsy), neuroinflammatory diseases (e.g., meningitis, encephalitis), neuromuscular disorders (e.g., amyotrophic lateral sclerosis, myasthenia gravis), and congenital neurological disorders (e.g., spina bifida, cerebral palsy).

6) Reproductive system disease
Reproductive system disease is any disease involving the organs and structures of the reproductive system. Reproductive system diseases constitute a class of diseases that include: male reproductive diseases (e.g., erectile dysfunction, prostatitis, testicular cancer), female reproductive diseases (e.g., endometriosis, ovarian cancer), sexually transmitted infections (e.g., chlamydia, gonorrhea, syphilis), reproductive organ malformations (e.g., uterine fibroids, congenital absence of the vas deferens), and conditions affecting fertility and pregnancy (e.g., infertility, ectopic pregnancy, preeclampsia).

7) Circulatory system disease
The circulatory system of the human body is a complex system consisting of the heart, blood vessels, and blood. It is responsible for transporting oxygen, nutrients, hormones, and metabolic waste products to maintain normal bodily functions. Here, the circulatory system diseases include blood system diseases and non-cardiovascular diseases of the circulatory system. We list some examples below: anemia, leukemia, lymphoma, hemophilia, sickle cell anemia, aplastic anemia, myelodysplastic syndromes, chronic myeloid leukemia, idiopathic thrombocytopenic purpura, thrombotic thrombocytopenic purpura, thalassemia, multiple myeloma, chronic lymphocytic leukemia, acute lymphoblastic leukemia, aortic aneurysm, atherosclerosis, peripheral artery disease, venous thromboembolism, varicose veins, Raynauds disease, vasculitis, lymphedema, lymphadenitis, and lymphangitis.

8) Skin disease
Skin disease is any disease involving the integumentary system, which includes the skin, hair, nails, and related glands. Skin diseases constitute a class of disorders that encompass dermatological conditions such as inflammatory skin disorders (e.g., eczema, psoriasis),

infectious skin diseases (e.g., bacterial infections like cellulitis, viral infections like herpes), allergic skin conditions (e.g., contact dermatitis, hives), autoimmune skin disorders (e.g., vitiligo, pemphigus), skin cancers (e.g., melanoma, basal cell carcinoma), genetic skin disorders (e.g., ichthyosis, epidermolysis bullosa), and disorders affecting the appendages of the skin (e.g., alopecia, nail disorders).

9) Orthopedic disease
   Orthopedic disease is any disease involving the musculoskeletal system, which includes the bones, joints, muscles, ligaments, tendons, and related tissues. Orthopedic diseases constitute a class of disorders that encompass conditions such as degenerative joint diseases (e.g., osteoarthritis), inflammatory joint diseases (e.g., rheumatoid arthritis), fractures and trauma affecting bones and joints, congenital musculoskeletal disorders (e.g., congenital hip dysplasia), metabolic bone diseases (e.g., osteoporosis), musculoskeletal tumors (e.g., osteosarcoma, chondrosarcoma), connective tissue disorders affecting joints and ligaments (e.g., Ehlers-Danlos syndrome), and disorders requiring orthopedic surgery (e.g., joint replacements, spinal surgeries).

Considering that the liver's primary functions are metabolic functions, such as the metabolism of carbohydrates, lipids, and proteins, as well as detoxification functions, such as breaking down and removing toxins and drugs from the body and converting them into non-toxic or easily excretable forms, most liver-related diseases are classified under the digestive system.

Considering that the kidney's primary functions are to excrete metabolic waste products such as urea, uric acid, and toxins, regulate water and electrolyte balance, and maintain acid-base balance, most kidney-related diseases are classified under the circulatory system. However, if the disease involves the kidney's hormonal regulation functions, it is classified as an endocrine disorder.

Infectious diseases are not listed as a separate department. Instead, they are classified into one of the nine clinical specialties based on the organs affected by the disease. For example, septicemia affects the blood system, so it is classified under circulatory system diseases; purulent meningitis affects the nervous system, so it is classified under the nervous system.

Immune system diseases are also not listed as a separate department. They are classified into one of our nine clinical specialties based on the organs affected by the disease. For example, arthritis is classified under orthopedic diseases, while lupus is classified under skin diseases.

Annotation Process

The annotation process was completed by an annotation team from the University of Minnesota to generate the ground-truth on DDx, interpretation, and medical specialty. The annotation team, including two senior members and a junior member as annotators, carried out the annotation effort. Specifically, one senior annotator is a registered nurse with more than 15 years of experience (H.N.), the other senior annotator is a physician with a medical degree and clinical certification (B.Y.), and the junior annotator is a resident in the clinical department (J.L.). Following the same protocol, the team generated the ground-truth annotation for DDx, interpretation, and medical specialty, respectively.

The data annotation is an iterative process. First, two members (i.e., H.N. and J.L.) annotated 10 data instances independently, discussed the results, and compared the inter-annotator agreement (IAA)

score. If these two people have discrepancies in their annotations, they will discuss and ultimately reach an agreement. When the discrepancies were hard to resolve through discussions, a third physician (B.Y.) examined the case and made the final annotation. Second, the annotators worked on another 20 instances, calculated the IAA score of the results, and discussed for better consistency. Third, another 30 instances are used for training the annotators and evaluating their agreement on annotation. The above steps are repeated until all the data samples are annotated. In this way, the annotation team generated the final gold standard dataset.

We respectively examined the IAA score on DDx, interpretation, and specialty. Specifically, we used the Jaccard score to measure the IAA for DDx and interpretation. The value of the Jaccard score ranged from 0 to 1; the larger the value, the agreement was better. The results showed that the final IAA scores on DDx and interpretation were 0.885 and 0.859, showing a great agreement between the annotators. As for the specialty, we adopted Cohen's Kappa score (κ) to evaluate the IAA. In detail, we first considered one of the annotations as ground-truth and the other as predictions then rotated it and took the average score as the result. The final κ score on specialty was 0.914, showing a high consistency in the annotation of the clinical specialty.

Data Annotation

Differential Diagnosis

a. Narrative descriptions: a list of possible conditions that are most likely to cause a patient's symptoms.

b. All labels will be used: the names of diseases.

c. Annotation example:

(1) Example of a clinical note:

A 25-year-old male complains of left chest pain and LUQ pain following an MVA. The patient struck a tree with his car at a slow speed. The chest pain is 8110. It is exacerbated with movement or when he takes a deep breath, and nothing relieves it. He reports dyspnea and a productive cough with a low-grade fever but denies LOC, headache, change in mental status, or change in vision. No cardiovascular or neurologic symptoms. No nausea, vomiting, neck stiffness, or unusual fluid from the mouth or nose. No dysuria. His last meal was 5 hours ago. He denies being under the influence of alcohol or drugs. ROS: As per HPI. Allergies: NKDA. Medications: None. PMH: Infectious mononucleosis 2 months ago. PSH: None. SH: No smoking, occasional EtOH, no illicit drugs. FH: Noncontributory. Physical Examination Patient is in acute distress, dyspneic. VS: Temp 100°F, RR 22/minute. HEENT: Atraumatic, no JVD, no bruises, PERRLA, EOMI, no pharyngeal edema or exudates. Chest: Two large ecchymosis on left chest, left rib tenderness, decreased breath sounds over left lung field, right lung fields clear. Heart: RRR; S1/S2 WNL; no murmurs, rubs, or gallops. Abdomen: Soft, nondistended, BS x 4 quadrants, LUQ tenderness, no rebound or guarding, no organomegaly. Skin: No bruises or lacerations. Neuro: Mental status: Alert and oriented x 3. Cranial nerves: 2-12 grossly intact. Motor: Strength 515 in all muscle groups. Sensation: Intact to pinprick and soft touch.

(2) DDx annotation: ["Pneumothorax", "Hemothorax", "Pneumonia"].

DDx Interpretation

a. Narrative descriptions: denote the corresponding reasons or explanations that supported the potential diagnosis.

b. All labels will be used: the signs and symptoms that appeared in the patient's clinical note.

c. Annotation example:

(1) Example of a clinical note:

A 25-year-old male complains of left chest pain and LUQ pain following an MVA. The patient struck a tree with his car at a slow speed. The chest pain is 8110. It is exacerbated with movement or when he takes a deep breath, and nothing relieves it. He reports dyspnea and a productive cough with a low-grade fever but denies LOC, headache, change in mental status, or change in vision. No cardiovascular or neurologic symptoms. No nausea, vomiting, neck stiffness, or unusual fluid from the mouth or nose. No dysuria. His last meal was 5 hours ago. He denies being under the influence of alcohol or drugs. ROS: As per HPI. Allergies: NKDA. Medications: None. PMH: Infectious mononucleosis 2 months ago. PSH: None. SH: No smoking, occasional EtOH, no illicit drugs. FH: Noncontributory. Physical Examination Patient is in acute distress, dyspneic. VS: Temp 100°F, RR 22/minute. HEENT: Atraumatic, no JVD, no bruises, PERRLA, EOMI, no pharyngeal edema or exudates. Chest: Two large ecchymosis on left chest, left rib tenderness, decreased breath sounds over left lung field, right lung fields clear. Heart: RRR; S1/S2 WNL; no murmurs, rubs, or gallops. Abdomen: Soft, nondistended, BS x 4 quadrants, LUQ tenderness, no rebound or guarding, no organomegaly. Skin: No bruises or lacerations. Neuro: Mental status: Alert and oriented x 3. Cranial nerves: 2-12 grossly intact. Motor: Strength 515 in all muscle groups. Sensation: Intact to pinprick and soft touch.

(2) DDx interpretation annotation:

{Pneumothorax: [Left -sided chest pain following an MVA, Decreased breath sounds over left lung field, Pain is exacerbated by movement and deep breaths, RR 22/minute, Dyspnea, Bruising and tenderness over left chest], Hemothorax: [Left -sided chest pain following an MVA, Decreased breath sounds over left lung field, Dyspnea, RR 22/minute, Cough, Bruising and tenderness over left chest], Pneumonia: [Unilateral chest pain, Temperature 100 °*F* , Productive cough, RR 22/minute, Low - grade fever]}

Clinical Specialty

a. Narrative descriptions: is a branch of medical practice that is focused on a defined group of patients, diseases, skills, or philosophy.

b. All labels will be used: cardiovascular disease, digestive system disease, respiratory disease, endocrine disorder, nervous system disease, reproductive system disease, circulatory system disease, skin disease, and orthopedic disease.

c. Annotation example:

(1) Example of a clinical note:

A 25-year-old male complains of left chest pain and LUQ pain following an MVA. The patient struck a tree with his car at a slow speed. The chest pain is 8110. It is exacerbated with movement or when he takes a deep breath, and nothing relieves it. He reports dyspnea and a productive cough with a low-grade fever but denies LOC, headache, change in mental status, or change in vision. No cardiovascular or neurologic symptoms. No nausea, vomiting, neck stiffness, or unusual fluid from the mouth or nose. No dysuria. His last meal was 5 hours ago. He denies being under the influence of alcohol or drugs. ROS: As per HPI. Allergies: NKDA. Medications: None. PMH: Infectious mononucleosis 2 months ago. PSH: None. SH: No smoking, occasional EtOH, no illicit drugs. FH: Noncontributory. Physical Examination Patient is in acute distress, dyspneic. VS: Temp 100°F, RR 22/minute. HEENT: Atraumatic, no JVD, no bruises, PERRLA, EOMI, no pharyngeal edema or exudates. Chest: Two large ecchymosis on left chest, left rib tenderness, decreased breath sounds over left lung field, right lung fields clear. Heart: RRR; S1/S2 WNL; no murmurs, rubs, or gallops. Abdomen: Soft, nondistended, BS x 4 quadrants, LUQ tenderness, no rebound or guarding, no organomegaly. Skin: No bruises or lacerations. Neuro: Mental status: Alert and oriented x 3. Cranial nerves: 2-12 grossly intact. Motor: Strength 515 in all muscle groups. Sensation: Intact to pinprick and soft touch.

(2) Annotation of the clinical specialty: Respiratory disease.

Supplementary Appendix 2

In this section, we illustrate the details of automatic evaluation and human evaluation.

For the automatic evaluation, we assessed the consistency between the ground-truth diagnoses and the predictions generated by GPT-4o. We assumed that advanced LLMs possess sufficient medical knowledge to determine whether a ground-truth diagnosis and its corresponding prediction refer to the same disease. The specific prompts used for the LLM are provided in Supplementary Appendix 3. We considered diagnoses with different terminologies but equivalent meanings as correct predictions. For instance, “Breast Cancer” and “Breast Malignancy” or “Epilepsy” and “Seizure Disorder” were treated as the same diagnosis. Additionally, when one diagnosis encompassed another (e.g., a subtype), we judged them as similar and considered the prediction correct. For example, “Diabetes Mellitus” and “Type I Diabetes Mellitus” were treated as equivalent for this purpose. However, if the two diagnoses represented distinctly different medical conditions, the prediction was deemed incorrect. For instance, “Benign Breast Tumor” and “Breast Malignancy” were treated as separate diagnoses, as were “Type I Diabetes Mellitus” and “Type II Diabetes Mellitus.” Following this protocol, we evaluated the correctness of each diagnosis in the differential diagnosis (DDx) task and recorded the total number of correct predictions. Following related papers, we took accuracy as the primary metric for assessing diagnostic performance, i.e.,

$$\text{Diagnostic Accuracy} = \frac{\text{Cumulative number of correct diagnoses}}{\text{Total number of diagnoses}} \tag{1}$$

For automatic evaluation of interpretation performance, we employed metrics designed to assess the semantic alignment between the reference text and the predicted text, rather than relying solely on string matching. These metrics included accuracy, BERTScore, SentenceBert, and METEOR, which were widely used in related tasks. Concretely, the interpretation accuracy was computed as:

$$\text{Interpretation Accuracy} = \frac{\text{Cumulative number of correct interpretations}}{\text{Total number of interpretations}} \tag{2}$$

The prompts utilized to evaluate the semantic consistency of the ground-truth and predicted explanations are shown in Supplementary Appendix 3. The key point is to measure the consistency of semantic meaning. For instance, given the ground-truth of “a severe fever of 104°F”, a predicted explanation of “a severe fever” is considered correct, while another one, such as “dry cough”, is considered wrong. BERTScore employs the BERT model to determine the semantic similarity between reference and generated text, offering a context-aware evaluation of model performance. SentenceBert measures sentence similarity using a BERT model that generates dense vector representations, facilitating efficient and accurate semantic comparisons. METEOR assesses the harmonic mean of unigram precision and recall, utilizing stemmed forms and synonym equivalence.

In addition to the automatic evaluation, expert assessments were conducted to evaluate performance. For the diagnosis task, physicians assessed the consistency between predictions and ground-truth diagnoses, adhering to the same protocol described for automatic evaluation. For the generated explanations, we adopted qualitative metrics inspired by related works to evaluate performance across three dimensions: Correctness, Completeness, and Usefulness. The Correctness Score reflects the medical accuracy of the statements, while the Completeness Score measures the extent to which the explanation addresses symptom descriptions comprehensively. The Usefulness Score assesses the overall value of the explanation in aiding diagnosis. Each metric was rated on an integer scale from 1 to 5. A score of 1 indicates the lowest performance, with the following implications: more than 80% of the predicted explanations were irrelevant to the ground-truth (Correctness), over 80% of explanations were missing compared to the ground-truth (Completeness), and the explanations were considered entirely unhelpful (Usefulness). In contrast, a score of 5 denotes the highest performance, where less than 20% of predicted explanations were irrelevant to the ground-truth, the number of missing explanations is less than 20%, and the explanations were deemed highly useful. Each note was independently reviewed by two physicians, with any disagreements resolved by a third reviewer.

Notably, we randomly selected 100 notes for manual evaluation and only presented the results of the explanation in the manuscript (Fig 2(b)). In our study, manual evaluation focused on the three qualitative metrics, but the quantitative metric of Interpretation Accuracy could also be manually assessed. This would involve domain experts evaluating the semantic consistency between ground-truth and predicted explanations using the established protocol. Furthermore, the same evaluation protocol was uniformly applied across all methods to ensure fair performance comparisons. While future studies may choose to develop customized evaluation protocols for both diagnosis and explanation assessments, whether through human or LLM-based evaluations, we believe that altering the evaluation protocol would not affect the superiority of the proposed framework over the baselines, as all methods were evaluated using a unified standard.

<u>Supplementary Appendix 3</u>

Below are the prompts used for the GPT-4o to compare the ground-truth diagnosis and interpretation with predictions from models, respectively.

Prompts for diagnosis comparison:

"You are an experienced doctor. Please determine whether {key_pred} and {key_gnd} refer to the same disease. Please note, do not simply match the text.

From a medical perspective, if they are the same or nearly the same disease, or if {key_pred} is a subset of {key_gnd}, your response should be {'1'}.

From a medical perspective, if they are essentially different diseases, your response should be {'0'}."

Prompts for interpretation comparison:

"You are an experienced doctor. Please determine whether {reason_i} and {reason_j} are basically describing the same symptoms (signs).

From a medical perspective, if both descriptions of the patient's symptoms are roughly the same or closely related, return {'1'}. From a medical perspective, if the conditions (signs) or medical examination results described in these two texts have a substantial amount of content that is basically similar, return {'1'}.

From a medical perspective, if both descriptions of the patient's symptoms are different, return {'0'}.

Please note, do not simply match the text. From a doctors perspective, assessing whether both descriptions are referring to the same symptoms."

Supplementary Appendix 4

Below is the differential diagnosis performance of the methods implemented with Llama3-70B and BioLlama3-70B. Notably, the performance built on GPT-4 and GPT-4o is shown in Figure 1(b) of the manuscript.

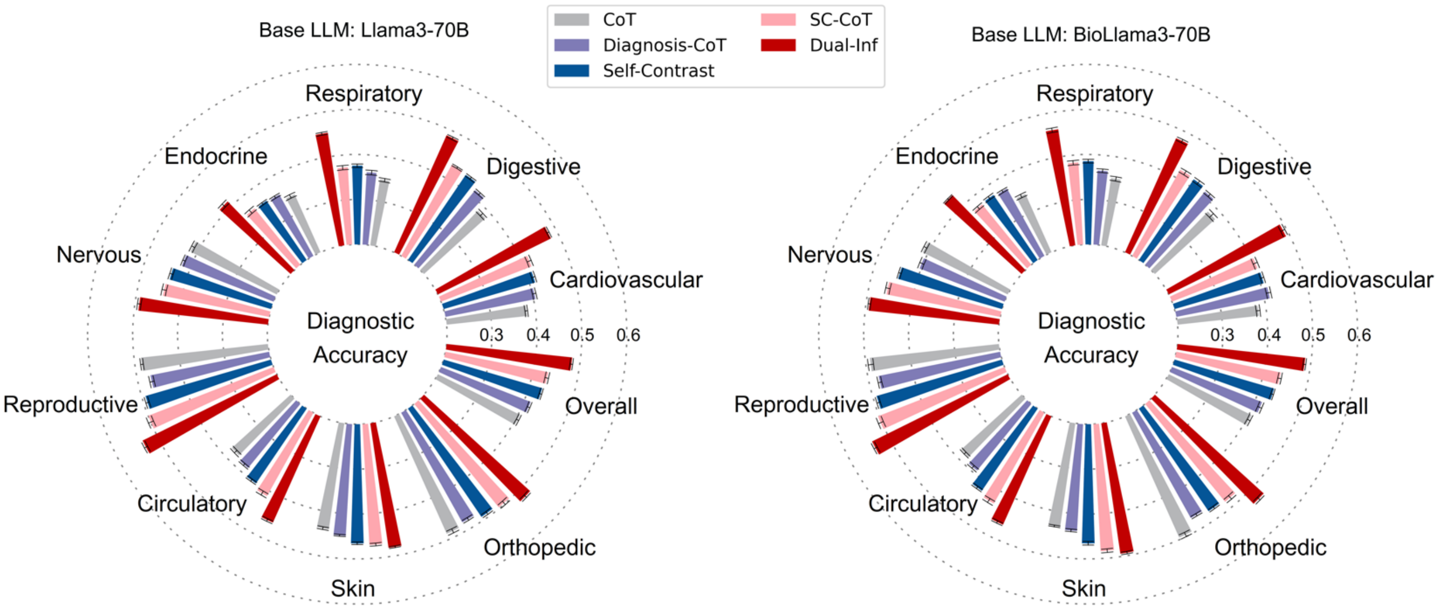

Supplementary Figure 5. Differential diagnosis performance built on two base LLMs (Llama3-70B and BioLlama3-70B) over nine specialties. The results are averaged over five runs. Standard deviations are also shown.

Supplementary Appendix 5

Below is the interpretation performance of the methods implemented with GPT-4, GPT-4o, Llama3-70B, and BioLlama3-70B. The metrics included Interpretation Accuracy, BERTScore, SentenceBert, and METEOR. Notably, partial results on GPT-4 are shown in Figure 2(a) of the manuscript; we present the rest of the results on SentenceBert and METEOR as follows.

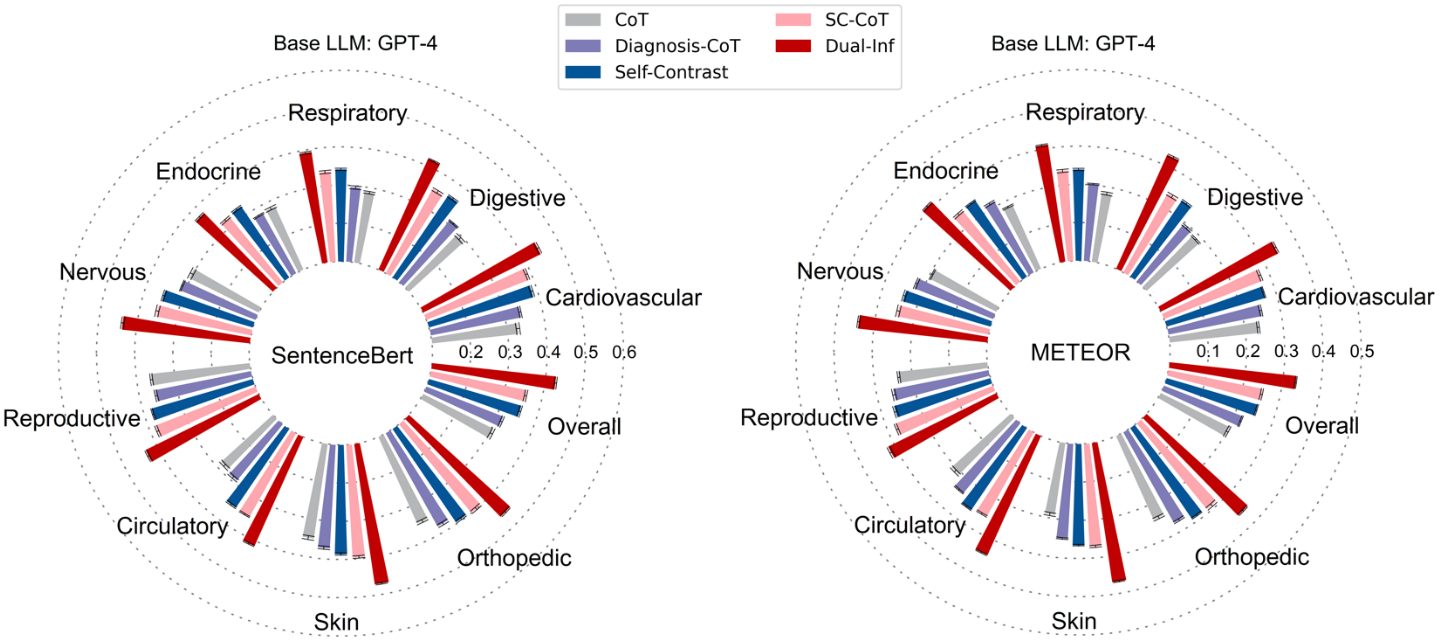

Supplementary Figure 6. Interpretation performance w.r.t SentenceBert and METEOR across nine clinical specialties. The methods are implemented with GPT-4. The results are averaged over five runs. Standard deviations are also shown.

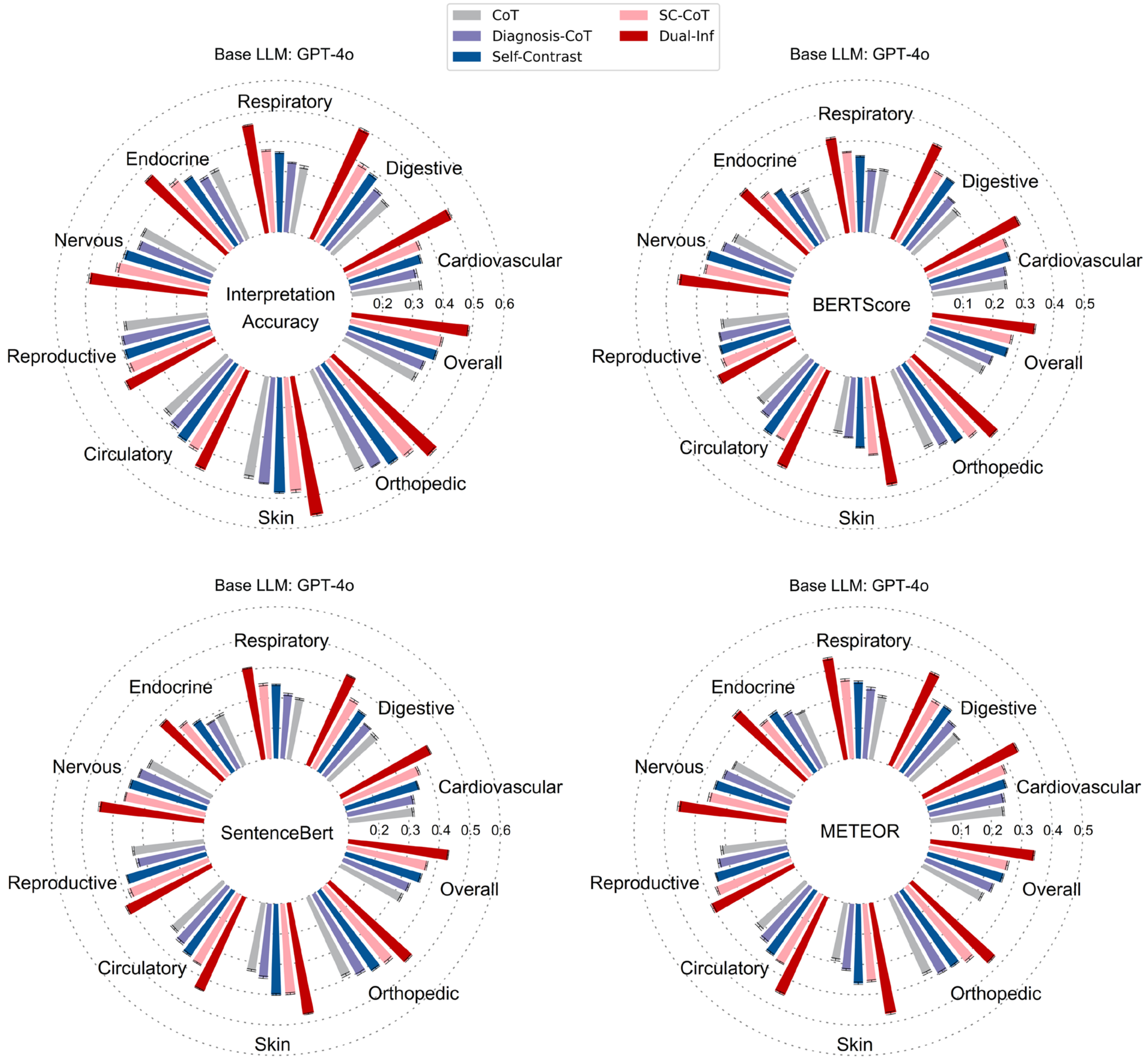

Supplementary Figure 7. Interpretation performance w.r.t four metrics, interpretation accuracy (see Eq. 2), BERTScore, SentenceBert, and METEOR across nine clinical specialties. The methods are implemented with GPT-4o. The results are averaged over five runs. Standard deviations are also shown.

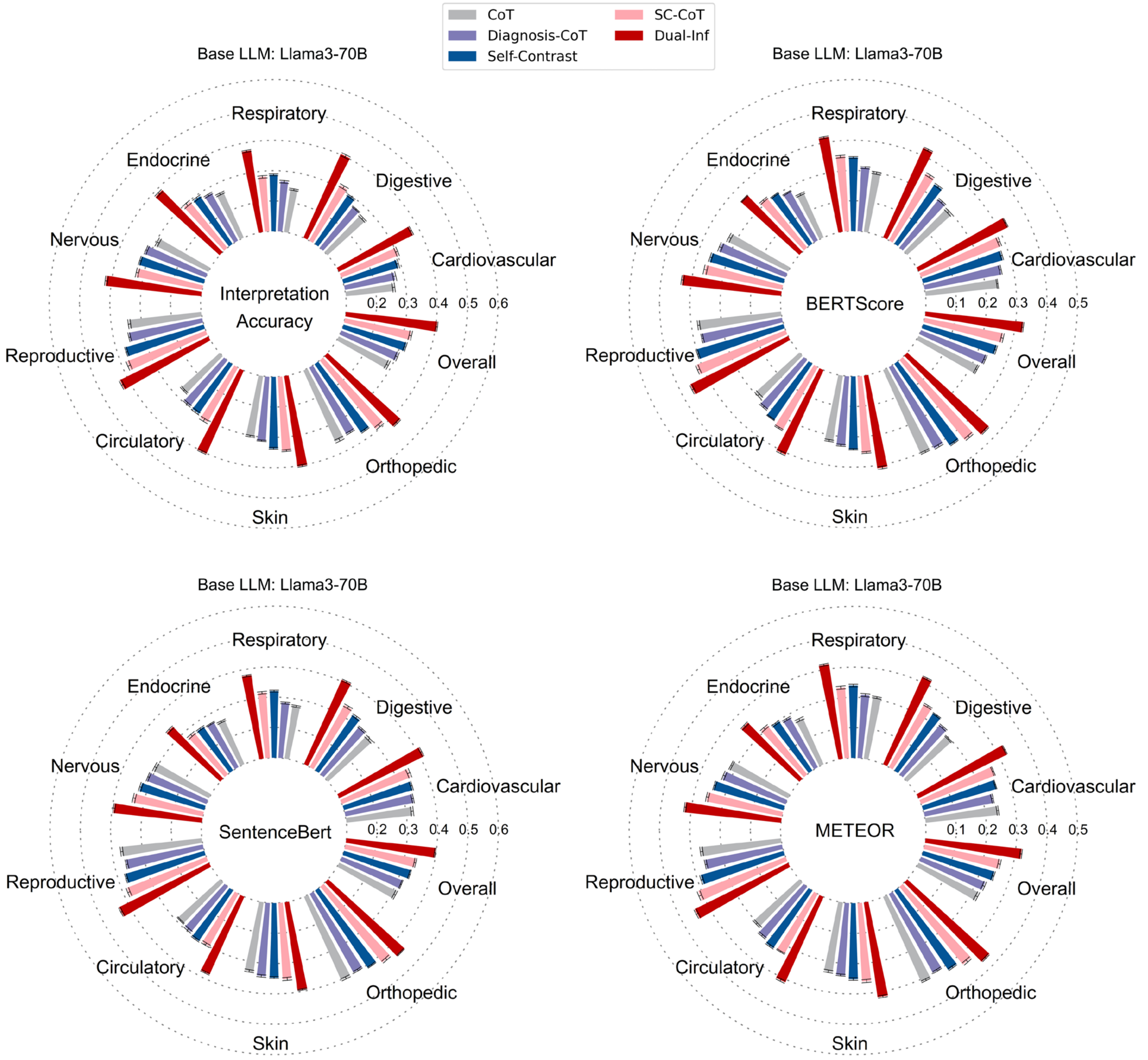

Supplementary Figure 8. Interpretation performance w.r.t four metrics, interpretation accuracy (see Eq. 2), BERTScore, SentenceBert, and METEOR across nine clinical specialties. The methods are implemented with Llama3-70B. The results are averaged over five runs. Standard deviations are also shown.

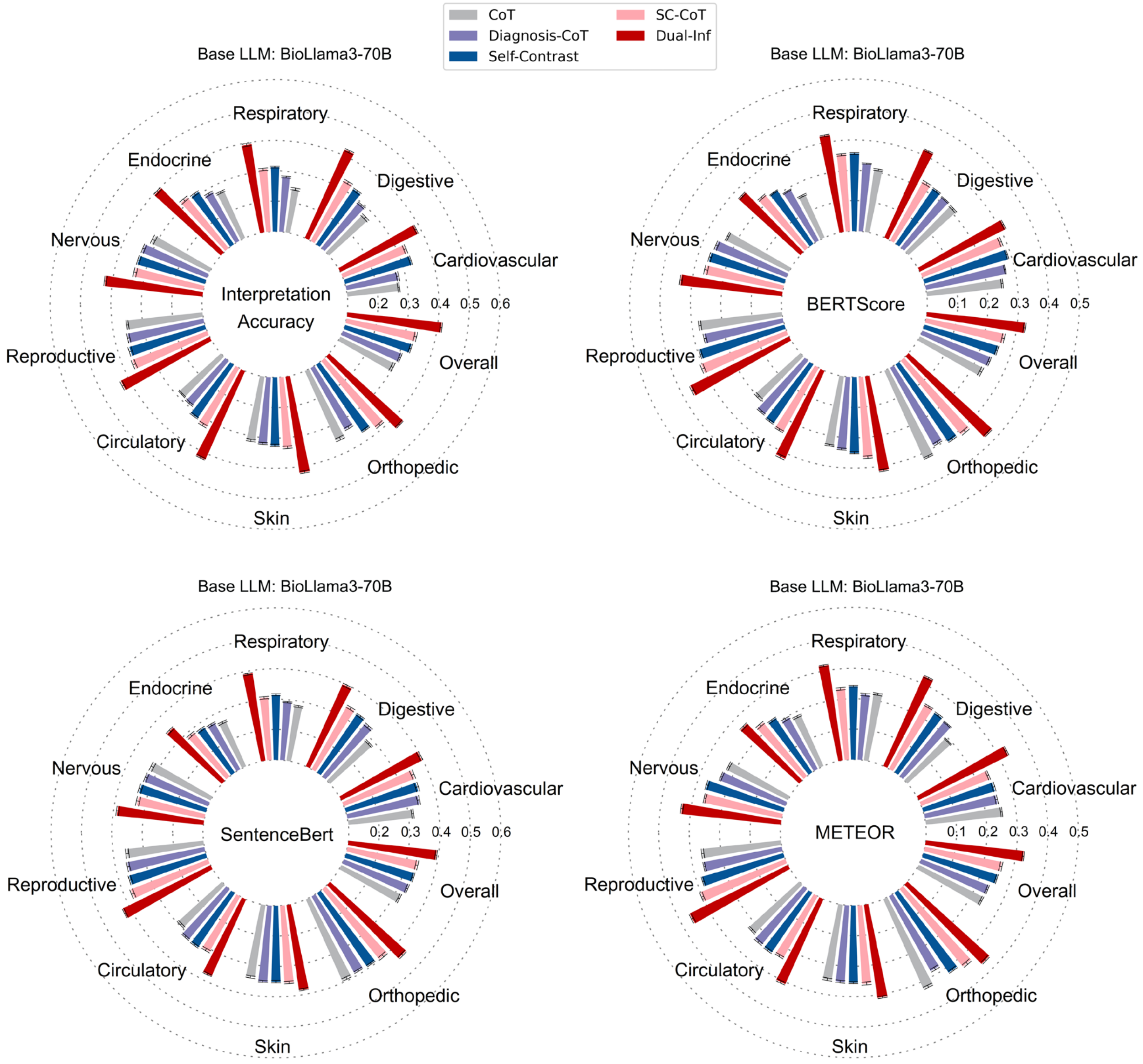

Supplementary Figure 9. Interpretation performance w.r.t four metrics, interpretation accuracy (see Eq. 2), BERTScore, SentenceBert, and METEOR across nine clinical specialties. The methods are implemented with BioLlama3-70B. The results are averaged over five runs. Standard deviations are also shown.

Supplementary Appendix 6

Below are the error analysis results of the methods in each clinical specialty. The results demonstrated that the errors fell into all the specialties, while the nervous and digestive diseases had more errors. This was because the two specialties had more samples in our dataset. After dividing the error count by the total sample in the specialty, each specialty generally had similar error counts.

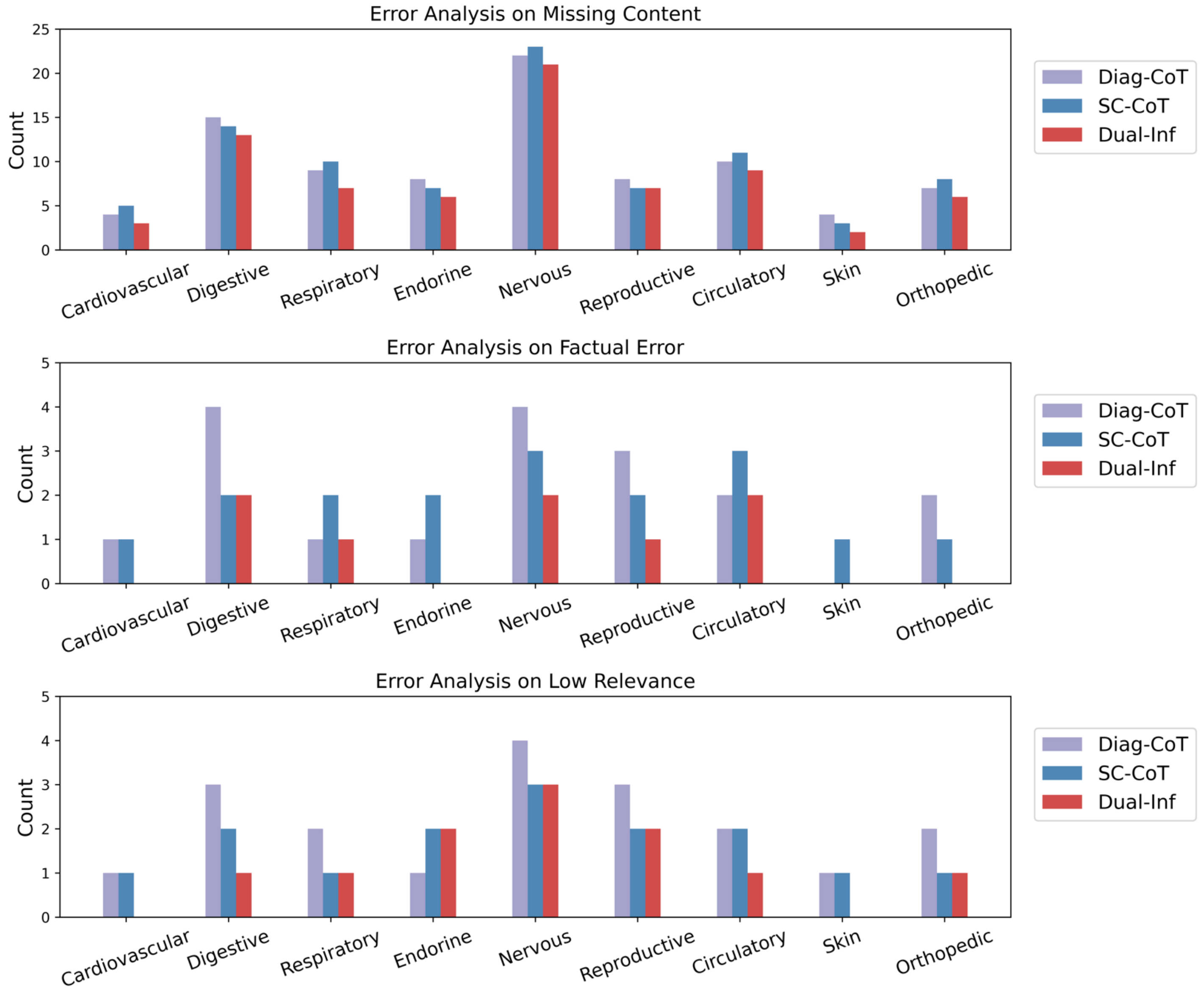

Supplementary Figure 10. Error analysis on interpretation in each clinical specialty. The methods are implemented with GPT-4. We manually examined 100 cases, recorded the count of the error type, and presented the results of one run. Diag-CoT denotes Diagnosis-CoT.

Supplementary Appendix 7

Below is a case study that presents the prediction results of Dual-Inf after each iteration. We implemented Dual-Inf by taking GPT-4 as the base LLM, assigning the maximum iteration number as 5, and setting the threshold β as 3. The results demonstrated that the iterative reflection mechanism in Dual-Inf helped to boost diagnostic accuracy and interpretation performance. Specifically, as *Hypokalemia, Medication-induced side effects,* and *Renal artery stenosis* are low-confidence diagnoses after the first iteration, it turned back to the forward-inference module for another prediction. By taking these low-confidence diagnoses as feedback, Dual-Inf had one more correct diagnosis (i.e., *Primary hypertension*) and interpretation (i.e., *Blood pressure is 174/111 mmHg*) after the second iteration.

Notably, in our study, there are some cases where a false negative prediction is taken as feedback to the forward-inference module. This phenomenon happens when the backward-inference module recalls a limited number of associated signs or symptoms, or the examination module considers it a low-confidence diagnosis due to lacking sufficient evidence (explanations). Considering this, we carefully designed the prompts for the forward-inference module to enable the LLM to hold an objective attitude toward the feedback from the examination module, which mitigated the potential risk of a false negative error hindering correct prediction.

Supplementary Table 3. Prediction results of Dual-Inf after each iteration. Correct predictions are highlighted in yellow, and the results that differ from those of the previous iteration are highlighted in blue.

Patient's symptom description:
A 56-year-old man with a history of hypertension presents to his physician with progressive fatigue and new onset muscle cramps. He has had no recent changes to his medication regimen, which includes hydrochlorothiazide, lisinopril, and amlodipine. His temperature is 98.0 °F (36.7 °C), blood pressure is 174/111 mmHg, pulse is 70/min, respirations are 12/min, and oxygen saturation is 98% on room air. The patients cardiopulmonary and abdominal exams are unremarkable. Laboratory values are ordered as seen below. Serum: Na+: 138 mEq/L Cl-: 100 mEq/L K+: 3.3 mEq/L HCO3-: 33 mEq/L BUN: 20 mg/dL Glucose: 129 mg/dL.

Ground-truth DDx:
Essential (primary) hypertension, Secondary hypertension, Renovascular disease, Primary aldosteronism, Cushings syndrome , Pheochromocytoma.

Ground-truth interpretation:
The patient may suffer from *Essential (primary) hypertension* because of the following symptoms or evidence:
a history of hypertension, blood pressure is 174/111 mmHg

The patient may suffer from *Secondary hypertension* because of the following symptoms or evidence:
progressive fatigue , new onset muscle cramps, blood pressure is 174/111 mmHg, K+: 3.3 mEq/L

The patient may suffer from *Renovascular disease* because of the following symptoms or evidence:
progressive fatigue , new onset muscle cramps, blood pressure is 174/111 mmHg

The patient may suffer from *Primary aldosteronism* because of the following symptoms or evidence:
progressive fatigue , new onset muscle cramps, K+: 3.3 mEq/L, HCO3 -: 33 mEq/L

The patient may suffer from *Cushings syndrome* because of the following symptoms or evidence:

progressive fatigue, new onset muscle cramps, blood pressure is 174/111 mmHg, K+: 3.3 mEq/L, Glucose: 129 mg/dL

The patient may suffer from *Pheochromocytoma* because of the following symptoms or evidence:
progressive fatigue, new onset muscle cramps

Prediction results of Dual-Inf (GPT-4) after the 1st iteration

Predicted DDx:
Secondary hypertension, Hypokalemia, Cushings syndrome, Chronic kidney disease, Medication-induced side effects, Renal artery stenosis

Predicted interpretation:
The patient may suffer from *Secondary hypertension* because of the following symptoms or evidence:
Irregular heartbeat, Progressive fatigue, Blood pressure is 174/111 mmHg

The patient may suffer from *Hypokalemia* because of the following symptoms or evidence:
K+: 3.3 mEq/L , Respirations are 12/min

The patient may suffer from *Cushings syndrome* because of the following symptoms or evidence:
Blood pressure is 174/111 mmHg , Fatigue , Glucose: 129 mg/dL , K+: 3.3 mEq/L

The patient may suffer from *Chronic kidney disease* because of the following symptoms or evidence:
Fatigue , Blood pressure is 174/111 mmHg , BUN: 20 mg/dL , K+: 3.3 mEq/L

The patient may suffer from *Medication-induced side effects* because of the following symptoms or evidence:
Use of hydrochlorothiazide , Blood pressure is 174/111 mmHg

The patient may suffer from *Renal artery stenosis* because of the following symptoms or evidence:
BUN: 20 mg/dL , Progressive fatigue

Prediction results of Dual-Inf (GPT-4) after the 2nd iteration

Predicted DDx:
Secondary hypertension, Primary hypertension, Cushings syndrome, Chronic kidney disease, Hypokalemia, Renal artery stenosis

Predicted interpretation:
The patient may suffer from *Secondary hypertension* because of the following symptoms or evidence:
Irregular heartbeat, Progressive fatigue, Blood pressure is 174/111 mmHg

The patient may suffer from *Primary hypertension* because of the following symptoms or evidence:
Use of hydrochlorothiazide , Blood pressure is 174/111 mmHg

The patient may suffer from *Cushings syndrome* because of the following symptoms or evidence:
Blood pressure is 174/111 mmHg , Fatigue , Glucose: 129 mg/dL , K+: 3.3 mEq/L

The patient may suffer from *Chronic kidney disease* because of the following symptoms or evidence:
Fatigue , Blood pressure is 174/111 mmHg , BUN: 20 mg/dL

The patient may suffer from *Hypokalemia* because of the following symptoms or evidence:
K+: 3.3 mEq/L , Respirations are 12/min

The patient may suffer from *Renal artery stenosis* because of the following symptoms or evidence:
BUN: 20 mg/dL , Progressive fatigue , Blood pressure is 174/111 mmHg

Prediction results of Dual-Inf (GPT-4) after the 3rd iteration

Predicted DDx:
Secondary hypertension, Primary hypertension, Cushings syndrome , Chronic kidney disease, Renal artery stenosis, Metabolic alkalosis

Predicted interpretation:
The patient may suffer from *Secondary hypertension* because of the following symptoms or evidence:
Irregular heartbeat, Progressive fatigue, Blood pressure is 174/111 mmHg

The patient may suffer from *Primary hypertension* because of the following symptoms or evidence:
Use of hydrochlorothiazide , Blood pressure is 174/111 mmHg

The patient may suffer from *Cushings syndrome* because of the following symptoms or evidence:
Blood pressure is 174/111 mmHg , Fatigue , Glucose: 129 mg/dL , K+: 3.3 mEq/L

The patient may suffer from *Chronic kidney disease* because of the following symptoms or evidence:
Fatigue , Blood pressure is 174/111 mmHg , BUN: 20 mg/dL

The patient may suffer from *Renal artery stenosis* because of the following symptoms or evidence:
BUN: 20 mg/dL , Progressive fatigue , Blood pressure is 174/111 mmHg

The patient may suffer from *Metabolic alkalosis* because of the following symptoms or evidence:
Use of hydrochlorothiazide , HCO3 -: 33 mEq/L

Prediction results of Dual-Inf (GPT-4) after the 4th iteration

Predicted DDx:
Secondary hypertension, Primary aldosteronism, Cushings syndrome, Chronic kidney disease, Renal artery stenosis, Hypokalemia
Predicted interpretation:
The patient may suffer from *Secondary hypertension* because of the following symptoms or evidence:
Irregular heartbeat, Progressive fatigue, Blood pressure is 174/111 mmHg

The patient may suffer from *Primary aldosteronism* because of the following symptoms or evidence:
K+: 3.3 mEq/L, HCO3 -: 33 mEq/L , Progressive fatigue , Blood pressure is 174/111 mmHg

The patient may suffer from *Cushings syndrome* because of the following symptoms or evidence:
Blood pressure is 174/111 mmHg , Fatigue , Glucose: 129 mg/dL , K+: 3.3 mEq/L

The patient may suffer from *Chronic kidney disease* because of the following symptoms or evidence:
Fatigue , Blood pressure is 174/111 mmHg , BUN: 20 mg/dL

The patient may suffer from *Renal artery stenosis* because of the following symptoms or evidence:
BUN: 20 mg/dL , Progressive fatigue , Blood pressure is 174/111 mmHg

The patient may suffer from *Hypokalemia* because of the following symptoms or evidence:
K+: 3.3 mEq/L , Respirations are 12/min

Prediction results of Dual-Inf (GPT-4) after the 5th iteration

Predicted DDx:
Secondary hypertension, Primary aldosteronism, Cushings syndrome, Chronic kidney disease, Renal artery stenosis, Hypokalemia
Predicted interpretation:
The patient may suffer from *Secondary hypertension* because of the following symptoms or evidence:
Irregular heartbeat, Progressive fatigue, Blood pressure is 174/111 mmHg

The patient may suffer from *Primary aldosteronism* because of the following symptoms or evidence:
K+: 3.3 mEq/L, HCO3 -: 33 mEq/L , Progressive fatigue , Blood pressure is 174/111 mmHg

The patient may suffer from *Cushings syndrome* because of the following symptoms or evidence:
Blood pressure is 174/111 mmHg , Fatigue , Glucose: 129 mg/dL , K+: 3.3 mEq/L

The patient may suffer from *Chronic kidney disease* because of the following symptoms or evidence:
Fatigue , Blood pressure is 174/111 mmHg , BUN: 20 mg/dL

The patient may suffer from *Renal artery stenosis* because of the following symptoms or evidence:
BUN: 20 mg/dL , Progressive fatigue , Blood pressure is 174/111 mmHg

The patient may suffer from *Hypokalemia* because of the following symptoms or evidence:
K+: 3.3 mEq/L , Respirations are 12/min , Progressive fatigue

Supplementary Appendix 8

Below is a case study to demonstrate the superior interpretability of the proposed framework. In this example, Diagnosis-CoT, SC-CoT, and Dual-Inf were implemented with GPT-4o. Unlike the case study in Supplementary Table 3, where Dual-Inf filtered out low-confidence diagnoses and used the iterative reflection mechanism for another prediction, this example showcased that Dual-Inf directly generated the diagnoses and explanations. This happened as the number of explanations in the initial diagnoses exceeded the threshold β in the examination module.

The example in Supplementary Table 4 demonstrated that the three methods made two correct diagnoses, i.e., *Rheumatoid arthritis* and *Systemic lupus erythematosus,* and had one erroneous prediction. We also observed that, although SC-CoT had more explanations for the diagnoses than Diagnosis-CoT, the explanation was not aligned with the ground-truth. In contrast, the proposed framework made more accurate interpretations for the diagnoses, e.g., *Spontaneous abortions* for the diagnosis of *Systemic lupus erythematosus.* In short, the results verified the superior interpretability of the proposed framework for explainable differential diagnosis.

Supplementary Table 4. Case study of Diagnosis-CoT, SC-CoT, and Dual-Inf. The methods are implemented by taking GPT-4o as the base LLM. Correct predictions are highlighted in blue.

Patient's symptom description:
HPI: 33-year female complains of left knee pain that started 2 days ago and is causing difficulty in walking. She has swelling and redness in her left knee and a mild fever; but no chills. She denies trauma. She has a history of fatigue and painful wrists and fingers and has experienced 1-hour morning stiffness over the past 6 months. She also recalls multiple oral ulcers that resolved last month. She describes Raynaud phenomenon, but denies rash, photosensitivity, hair loss, or recent tick bites. She recalls a 10-lb weight loss over the past 6 months and has no appetite. ROS: Negative except as above. Allergies: NKDA. Medications: Tylenol. PMH: Episode of acute left big toe arthritis 5 years ago; gonorrhea 1 year ago. PSH: Two C-sections, 2 spontaneous abortions. SH: 1 pack per day for 10 years. Usually drinks 2-4 beers/week; on weekends drinks more; last ingestion 4 days ago; CAGE 0/4. No illicit drugs. Sexually active with multiple male partners; inconsistent condom use. FH: Mother has rheumatoid arthritis and lives in a nursing home. Physical Examination Patient is in no acute distress but favors the left knee. VS: WNL except for low-grade fever. HEENT: No oral lesions. Chest: Clear breath sounds bilaterally. Heart: RRR; S1/S2 WNL; no murmurs, rubs, or gallops. Abdomen: Soft, nondistended, BS x 4, no hepatosplenomegaly. Extremities: Erythema, tenderness, pain, and restricted range of motion on flexion and extension of left knee compared to right knee. swelling at left knee. Fingers and hands with stiffness bilaterally. Shoulder, elbow, wrist, hip, and ankle joints WNL bilaterally.

Ground-truth DDx:
Gout, Rheumatoid arthritis, Systemic lupus erythematosus

Ground-truth interpretation:
The patient may suffer from *Gout* because of the following symptoms or evidence:
Monoarticular joint pain and tenderness, History of swollen toe, Occasional alcohol use, Joint tenderness and stiffness, Swelling at left knee

The patient may suffer from *Rheumatoid arthritis* because of the following symptoms or evidence:
Morning joint stiffness, Family history of rheumatoid arthritis, fatigue, fever, multiple joint involvement, duration > 6 weeks, Joint tenderness and stiffness

The patient may suffer from *Systemic lupus erythematosus* because of the following symptoms or evidence:

Systemic symptoms (anorexia, weight loss, fatigue), History of multiple oral ulcers, History of 2 spontaneous abortions, Raynaud phenomenon, Joint tenderness and stiffness
**Diagnosis-CoT (GPT-4o) - predicted DDx:**<br>*Rheumatoid arthritis, Systemic lupus erythematosus, Septic arthritis*<br><br>**Diagnosis-CoT (GPT-4o) - predicted interpretation:**<br>The patient may suffer from *Rheumatoid arthritis* because of the following symptoms or evidence:<br>*History of fatigue and painful wrists and fingers, 1 -hour morning stiffness over the past 6 months, Family history of rheumatoid arthritis*<br><br>The patient may suffer from *Systemic lupus erythematosus* because of the following symptoms or evidence:<br>*Multiple oral ulcers, Raynaud phenomenon, 10 -lb weight loss over the past 6 months*<br><br>The patient may suffer from *Septic arthritis* because of the following symptoms or evidence:<br>*Left knee pain with swelling and redness, Mild fever, Difficulty in walking*
**SC-CoT (GPT-4o) - predicted DDx:**<br>*Rheumatoid arthritis, Systemic lupus erythematosus, Septic arthritis*<br><br>**SC-CoT (GPT-4o) - predicted interpretation:**<br>The patient may suffer from *Rheumatoid arthritis* because of the following symptoms or evidence:<br>*History of fatigue and painful wrists and fingers, 1 -hour morning stiffness over the past 6 months, Family history of rheumatoid arthritis*<br><br>The patient may suffer from *Systemic lupus erythematosus* because of the following symptoms or evidence:<br>*Multiple oral ulcers, Raynaud phenomenon, 10 -lb weight loss over the past 6 months, No appetite*<br><br>The patient may suffer from *Septic arthritis* because of the following symptoms or evidence:<br>*Left knee pain with swelling and redness, Mild fever, Difficulty in walking, No history of trauma*
**Dual-Inf (GPT-4o) - predicted DDx:**<br>*Rheumatoid arthritis, Systemic lupus erythematosus, Septic arthritis*<br><br>**Dual-Inf (GPT-4o) - predicted interpretation:**<br>The patient may suffer from *Rheumatoid arthritis* because of the following symptoms or evidence:<br>*History of fatigue and painful wrists and fingers, 1 -hour morning stiffness over the past 6 months, Family history of rheumatoid arthritis*<br><br>The patient may suffer from *Systemic lupus erythematosus* because of the following symptoms or evidence:<br>*Multiple oral ulcers, Raynaud phenomenon, Spontaneous abortions , 10 -lb weight loss over the past 6 months*<br><br>The patient may suffer from *Septic arthritis* because of the following symptoms or evidence:<br>*Left knee pain with swelling and redness, Mild fever, Difficulty in walking, No history of trauma*

Supplementary Appendix 9

Below are the prompts used for the three modules (i.e., forward-inference module, backward-inference module, and examination module) in the proposed Dual-Inference Large Language Model framework.

Prompts for the forward-inference module:

"You are an experienced doctor. Based on the provided patient information {patient_info_j} and the question {Question}, please make a diagnosis of the disease.

Below, I provide ten complete examples for you to understand the task and reference the response format.

Patient information: {example_patient_info_1} and the question: {Question} as well as the doctor's diagnoses and interpretation: {example_interpretation_1}.

Patient information: {example_patient_info_2} and the question: {Question} as well as the doctor's diagnoses and interpretation: {example_interpretation_2}.

…

Patient information: {example_patient_info_10} and the question: {Question} as well as doctor's diagnoses and interpretation: {example_interpretation_10}.

Please write your response in the corresponding format. Please note that the output should only be dictionary-type data.
Do not include any extraneous characters, such as 'python'; only output the content within the {{}}."

Question:

"Based on the patient's symptom description, please USE STEP-BY-STEP DEDUCTION TO INFER the most likely diagnoses. In other words, list the differential diagnosis. Please state the names of the most likely diseases. Additionally, for each possible disease, explain which pieces of patient information support this diagnosis. Please note that you should use STEP-BY-STEP DEDUCTION TO INFER the diagnoses and interpretations.

Please note that your response format should be a Python dictionary structure. This means the keys of the dictionary are the names of the predicted diseases, and the value corresponding to each key is the list of reasons supporting this prediction.

{

"Disease 1": ["reason 1", "reason 2", …, "reason k"],

"Disease 2": ["reason 1", "reason 2", …, "reason k"],

...

“Disease N”: [“reason 1”, “reason 2”, …, “reason k”]

}

Please note that your response should only be a dictionary.”

(The following prompts are added to the forward-inference module after receiving feedback from the examination module.)

“Your previous predicted diagnosis SEEMS WRONG. The differential diagnosis of the patient DOES NOT SEEM TO CONTAIN {low_confidence_diagnosis}. NOTABLY, THIS FEEDBACK MIGHT NOT NECESSARILY BE CORRECT. Please think twice and combine the following instructions to make another prediction.”

Prompts for the backward-inference module:

“You are an experienced doctor. Based on the provided disease name {diagnosis_info_j}, please USE STEP-BY-STEP DEDUCTION TO RECALL THE MEDICAL KNOWLEDGE YOU LEARNED AND LIST all the representative symptoms, physical examination results, and medical laboratory results for this disease.

Please note that the listed elements, e.g., symptoms or medical laboratory results, can support the diagnosis of the disease.

Please note that your output should have a specific format. Specifically, the output is a Python list object; each symptom in the list is an element. Each symptom should not contain double quotes internally but should be enclosed in double quotes at the beginning and end.

Again, please note that the output should only be in a Python list type; the elements of the list are related symptoms, lab test results, etc.

Do not include any extraneous characters, such as 'python'; only output the content within the {{}}.”

Prompts for the examination module:

(a) Prompts for checking the correctness of interpretations

“You are an experienced doctor. I need you to determine whether a symptom {disease_k_reason_j} belongs to the clinical manifestations or test results of the disease {disease_k}.

Specifically, please use your medical knowledge and the provided symptom list for the disease {disease_k} {disease_k_gnd_symptom_list} to determine if {disease_k_reason_j} also belongs to the clinical manifestations or test results of this disease. Please DO IT STEP-BY-STEP.

Below, I provide two complete examples for you to understand the task and reference the response format.

example_disease_symptom_list = [“Malaise”, “Loss of appetite”, “Bloating”, “Nausea”]

example_disease_symptom_A = ‘have a jaded appetite’

example_disease_symptom_B = ‘knee swelling and pain’

Based on medical knowledge and the symptom list of the disease {example_disease_symptom_list}, {example_disease_symptom_A} also belongs to the clinical manifestations or test results of this disease, so the output is ‘Yes’.

Based on medical knowledge and the symptom list of the disease {example_disease_symptom_list}, {example_disease_symptom_B} does not belong to the clinical manifestations or test results of this disease, so the output is ‘No’.

Please note that the output should be only 'Yes' or 'No'. Do not include any extraneous characters, such as ```; just output the two or three letters.”

(b) Prompts for checking the completeness of interpretations

eg_prompt_EHR_patient = “An 8-year-old boy is brought to the physician by his foster mother because of complaints from his teachers regarding poor performance at school for the past 8 months. He does not listen to their instructions, often talks during class, and rarely completes his school assignments. He does not sit in his seat in the classroom and often cuts in line at the cafeteria. His foster mother reports that he runs around a lot inside the house and refuses to help his sister with chores and errands. He frequently interrupts his foster mother’s conversations with others and talks excessively. She has found him trying to climb on the roof on multiple occasions. He was placed in foster care because of neglect by his biological parents 3 years ago. Physical examination shows no abnormalities. Neurologic examination shows no focal findings. Mental status examination shows a neutral affect.”

eg_prompt_disease_symptom_A = “talks excessively”

eg_prompt_disease_symptom_B = “do noy follow parent’s words”

eg_prompt_disease_symptom_C = “Low-grade fever”

eg_prompt_disease_symptom_D = “A flushed face”

“You are an experienced doctor. I need you to determine whether the patient’s condition description (electronic health record) {patient_EHR} mentions the clinical manifestations or test results of the disease {disease_k}.

Specifically, please use your medical knowledge to determine if a symptom of the disease {disease_k} {disease_k_reason_j} appears in this patient’s condition description {patient_EHR}. In other words, does the patient’s condition description {patient_EHR} mention this symptom {disease_k_reason_j}? Please USE STEP-BY-STEP DEDUCTION TO DO IT.

Please note, this is not asking for an exact word-for-word match, but rather whether the patient's condition description {patient_EHR} includes clinical manifestations similar to {disease_k_reason_j}.

Below, I provide several complete examples for you to understand the task and reference the response format.

Based on medical knowledge, a common symptom of a disease {eg_prompt_disease_symptom_A} appears in this patient's condition description {eg_prompt_EHR_patient}, so the output is 'Yes'.

Based on medical knowledge, a common symptom of a disease {eg_prompt_disease_symptom_B} appears in this patient's condition description {eg_prompt_EHR_patient}, so the output is 'Yes'.

Based on medical knowledge, a common symptom of a disease {eg_prompt_disease_symptom_C} does not appear in this patient's condition description {eg_prompt_EHR_patient}, so the output is 'No'.

Based on medical knowledge, a common symptom of a disease {eg_prompt_disease_symptom_D} does not appear in this patient's condition description {eg_prompt_EHR_patient}, so the output is 'No'.

Please note that the output should be only 'Yes' or 'No'. Do not include any extraneous characters, such as ```; just output the two or three letters."

Supplementary Appendix 10

Below are the prompts used for the comparative methods in this study, i.e., CoT, Diagnosis-CoT, Self-Contrast, and SC-CoT.

Prompts for the CoT method:

"You are an experienced doctor. Based on the provided patient information {patient_info_j} and the question {Question}, please make a diagnosis of the disease.

Below, I provide ten complete examples for you to understand the task and reference the response format.

Patient information: {example_patient_info_1} and the question: {Question} as well as the doctor's diagnoses and interpretation: {example_interpretation_1}.

Patient information: {example_patient_info_2} and the question: {Question} as well as the doctor's diagnoses and interpretation: {example_interpretation_2}.

…

Patient information: {example_patient_info_10} and the question: {Question} as well as doctor's diagnoses and interpretation: {example_interpretation_10}.

Please write your response in the corresponding format."

Question:

"Based on the patient's symptom description, please USE STEP-BY-STEP DEDUCTION TO INFER the most likely diagnoses. In other words, list the differential diagnosis. Please state the names of the most likely diseases. Additionally, for each possible disease, explain which pieces of patient information support this diagnosis. Please note that you should use STEP-BY-STEP DEDUCTION TO INFER the diagnoses and interpretations.

Please note that your response format should be a Python dictionary structure. This means the keys of the dictionary are the names of the predicted diseases, and the value corresponding to each key is the list of reasons supporting this prediction.

{

"Disease 1": ["reason 1", "reason 2", …, "reason k"],

"Disease 2": ["reason 1", "reason 2", …, "reason k"],

...

"Disease N": ["reason 1", "reason 2", …, "reason k"]

}

Please note that your response should only be a dictionary."

Prompts for the Diagnosis-CoT method:

"You are an experienced doctor. Based on the provided patient information {patient_info_j} and the question {Question}, please make a diagnosis of the disease.

Below, I provide ten complete examples for you to understand the task and reference the response format.

Patient information: {example_patient_info_1} and the question: {Question} as well as the doctor's diagnoses and interpretation: {example_interpretation_1}.

Patient information: {example_patient_info_2} and the question: {Question} as well as the doctor's diagnoses and interpretation: {example_interpretation_2}.

…

Patient information: {example_patient_info_10} and the question: {Question} as well as doctor's diagnoses and interpretation: {example_interpretation_10}.

Please write your response in the corresponding format."

Question:

"Based on the patient's symptom description, please USE STEP-BY-STEP DEDUCTION TO CREATE A DIFFERENTIAL DIAGNOSIS AND THEN USE STEP-BY-STEP DEDUCTION TO DETERMINE THE CORRECT INTERPRETATION. In other words, list the differential diagnosis. Please state the names of the most likely diseases. Additionally, for each possible disease, explain which pieces of patient information support this diagnosis. Please note that you should USE STEP-BY-STEP DEDUCTION TO CREATE A DIFFERENTIAL DIAGNOSIS AND THEN USE STEP-BY-STEP DEDUCTION TO DETERMINE THE CORRECT INTERPRETATION.

Please note that your response format should be a Python dictionary structure. This means the keys of the dictionary are the names of the predicted diseases, and the value corresponding to each key is the list of reasons supporting this prediction.

{

"Disease 1": ["reason 1", "reason 2", …, "reason k"],

"Disease 2": ["reason 1", "reason 2", …, "reason k"],

...

“Disease N”: [“reason 1”, “reason 2”, …, “reason k”]

}

Please note that your response should only be a dictionary.”

Prompts for the Self-Contrast method:

Initial Response Stage:

“You are an experienced doctor. Based on the provided patient information {patient_info_j} and the question {Question}, please make a diagnosis of the disease.

Below, I provide ten complete examples for you to understand the task and reference the response format.

Patient information: {example_patient_info_1} and the question: {Question} as well as the doctor’s diagnoses and interpretation: {example_interpretation_1}.

Patient information: {example_patient_info_2} and the question: {Question} as well as the doctor’s diagnoses and interpretation: {example_interpretation_2}.

…

Patient information: {example_patient_info_10} and the question: {Question} as well as doctor’s diagnoses and interpretation: {example_interpretation_10}.

Please write your response in the corresponding format.”

Question:

“Based on the patient’s symptom description, PLEASE INFER THE MOST LIKELY DIAGNOSES. YOU ARE ENCOURAGED TO THINK DIFFERENTLY TOWARD GETTING THE CORRECT ANSWERS. In other words, list the differential diagnosis. Please state the names of the most likely diseases. Additionally, for each possible disease, explain which pieces of patient information support this diagnosis. Please note that you should THINK DIFFERENTLY AND INFER THE MOST LIKELY DIAGNOSES AND INTERPRETATIONS.

Please note that your response format should be a Python dictionary structure. This means the keys of the dictionary are the names of the predicted diseases, and the value corresponding to each key is the list of reasons supporting this prediction.

{

“Disease 1”: [“reason 1”, “reason 2”, …, “reason k”],

“Disease 2”: [“reason 1”, “reason 2”, …, “reason k”],

...

“Disease N”: [“reason 1”, “reason 2”, …, “reason k”]

}

Please note that your response should only be a dictionary.”

Revision Stage:

“You are an experienced doctor. Based on the provided patient information {patient_info_j} and the question {Question}, please make a diagnosis of the disease.

Below, I provide ten complete examples for you to understand the task and reference the response format.

Patient information: {example_patient_info_1} and the question: {Question} as well as the doctor’s diagnoses and interpretation: {example_interpretation_1}.

Patient information: {example_patient_info_2} and the question: {Question} as well as the doctor’s diagnoses and interpretation: {example_interpretation_2}.

…

Patient information: {example_patient_info_10} and the question: {Question} as well as doctor’s diagnoses and interpretation: {example_interpretation_10}.

Please write your response in the corresponding format.”

Question:

“Below is THE SUMMARIZED CHECKLIST THAT SHOWS THE DIFFERENCES IN THE DIAGNOSTIC PREDICTION FROM DIFFERENT DOCTORS. Checklist: {Checklist}. Please RE-EXAMINE THE DIFFERENCES IN DIAGNOSES AND INTERPRETATIONS and output your final diagnosis and interpretations.

Please note that your response format should be a Python dictionary structure. This means the keys of the dictionary are the names of the predicted diseases, and the value corresponding to each key is the list of reasons supporting this prediction.

{

“Disease 1”: [“reason 1”, “reason 2”, …, “reason k”],

“Disease 2”: [“reason 1”, “reason 2”, …, “reason k”],

...

“Disease N”: [“reason 1”, “reason 2”, …, “reason k”]

}

Please note that your response should only be a dictionary."

Prompts for the SC-CoT method:

"You are an experienced doctor. Based on the provided patient information {patient_info_j} and the question {Question}, please make a diagnosis of the disease.

Below, I provide ten complete examples for you to understand the task and reference the response format.

Patient information: {example_patient_info_1} and the question: {Question} as well as the doctor's diagnoses and interpretation: {example_interpretation_1}.

Patient information: {example_patient_info_2} and the question: {Question} as well as the doctor's diagnoses and interpretation: {example_interpretation_2}.

…

Patient information: {example_patient_info_10} and the question: {Question} as well as doctor's diagnoses and interpretation: {example_interpretation_10}.

Please write your response in the corresponding format."

Question:

"Based on the patient's symptom description, PLEASE USE STEP-BY-STEP DEDUCTION TO INFER THE MOST LIKELY DIAGNOSES. YOU ARE ENCOURAGED TO THINK DIFFERENTLY TOWARD GETTING THE CORRECT ANSWERS. In other words, list the differential diagnosis. Please state the names of the most likely diseases. Additionally, for each possible disease, explain which pieces of patient information support this diagnosis. Please note that you should THINK DIFFERENTLY AND USE STEP-BY-STEP DEDUCTION TO INFER THE MOST LIKELY DIAGNOSES AND INTERPRETATIONS.

Please note that your response format should be a Python dictionary structure. This means the keys of the dictionary are the names of the predicted diseases, and the value corresponding to each key is the list of reasons supporting this prediction.

{

"Disease 1": ["reason 1", "reason 2", …, "reason k"],

"Disease 2": ["reason 1", "reason 2", …, "reason k"],

...

“Disease N”: [“reason 1”, “reason 2”, …, “reason k”]

}

Please note that your response should only be a dictionary.”

Supplementary Appendix 11

Below are the results of the hyper-parameter analysis. We analyzed the impact of the main hyper-parameter, i.e., threshold β, on the performance.

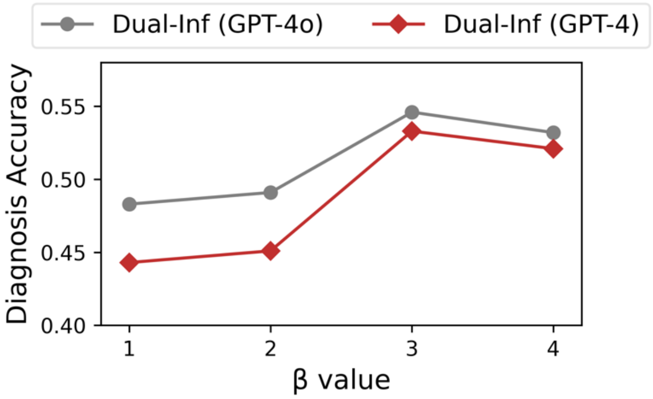

Supplementary Figure 11. The impact of the threshold β on diagnostic accuracy.

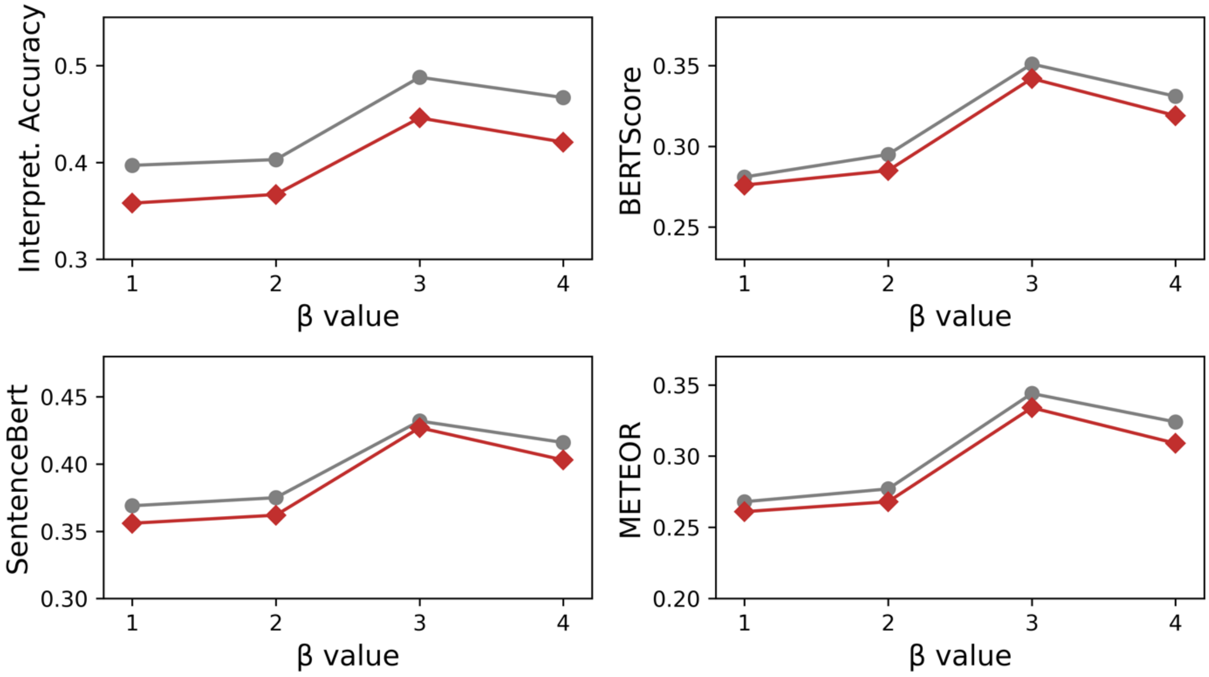

Supplementary Figure 12. The impact of the threshold β on explanation performance.

Supplementary Appendix 12

We further conducted an ablation study to analyze the impact of each component within Dual-Inf. Specifically, we built four method variants for comparison: (1) a method using only the forward-inference module (FI), (2) Dual-Inf without the backward-inference module (FI-EM), (3) FI-EM without the self-reflection mechanism (FI-EM*), and (4) Dual-Inf without the self-reflection mechanism (Dual-Inf*). We evaluated the models with automatic metrics, as shown in *Supplementary Appendix 2*. The results using GPT-4 as the base LLM are shown in Supplementary Table 5, and the results based on GPT-4o are presented in Supplementary Table 6. In Supplementary Table 5, we observed that the diagnostic accuracy of FI, FI-EM*, and Dual-Inf* was below 0.46, while FI-EM approached 0.5. Dual-Inf further improved diagnostic accuracy by approximately 7% compared to FI-EM. As for interpretability, FI and FI-EM* performed similarly across the four metrics. The interpretation accuracy of Dual-Inf* and FI-EM was around 0.358 and 0.373, respectively. Besides, Dual-Inf presented superior interpretability, with BERTScore boosting by 24% and METEOR by 28% over the Dual-Inf*.

Supplementary Table 5. Ablation study results with GPT-4.

Method	Diagnostic Performance	Interpretation Performance			
	Accuracy	Accuracy	BERTScore	SentenceBert	METEOR
FI	0.443 ± 0.008	0.294 ± 0.006	0.209 ± 0.004	0.307 ± 0.004	0.202 ± 0.003
FI-EM*	0.449 ± 0.006	0.302 ± 0.005	0.219 ± 0.002	0.320 ± 0.002	0.216 ± 0.002
FI-EM	0.497 ± 0.007	0.373 ± 0.007	0.265 ± 0.004	0.374 ± 0.003	0.286 ± 0.003
Dual-Inf*	0.453 ± 0.004	0.358 ± 0.003	0.276 ± 0.003	0.356 ± 0.003	0.261 ± 0.003
Dual-Inf	**0.533 ± 0.005**	**0.446 ± 0.004**	**0.343 ± 0.003**	**0.427 ± 0.002**	**0.335 ± 0.002**

Supplementary Table 6. Ablation study results with GPT-4o.

Method	Diagnostic Performance	Interpretation Performance			
	Accuracy	Accuracy	BERTScore	SentenceBert	METEOR
FI	0.472 ± 0.006	0.365 ± 0.005	0.217 ± 0.003	0.324 ± 0.003	0.219 ± 0.003
FI-EM*	0.478 ± 0.005	0.374 ± 0.004	0.228 ± 0.002	0.339 ± 0.003	0.231 ± 0.002
FI-EM	0.514± 0.006	0.416 ± 0.005	0.273 ± 0.004	0.387 ± 0.004	0.295 ± 0.002
Dual-Inf*	0.483 ± 0.004	0.397 ± 0.004	0.281 ± 0.003	0.369 ± 0.004	0.268 ± 0.003
Dual-Inf	**0.546 ± 0.005**	**0.488 ± 0.004**	**0.351 ± 0.004**	**0.432 ± 0.003**	**0.344 ± 0.002**
